# G Protein-Coupled Receptors (GPCRs) in Alzheimer’s Disease: A Focus on BACE1 Related GPCRs

**DOI:** 10.3389/fnagi.2016.00058

**Published:** 2016-03-24

**Authors:** Juan Zhao, Yulin Deng, Zhaotan Jiang, Hong Qing

**Affiliations:** ^1^School of Life Science, Beijing Institute of TechnologyBeijing, China; ^2^School of Physics, Beijing Institute of TechnologyBeijing, China

**Keywords:** G protein-coupled protein (GPCR), Alzheimer’s disease (AD), β-site APP cleaving enzyme 1 (BACE1), allosteric modulator, biased ligand

## Abstract

The G protein coupled receptors (GPCRs) have been considered as one of the largest families of validated drug targets, which involve in almost overall physiological functions and pathological processes. Meanwhile, Alzheimer’s disease (AD), the most common type of dementia, affects thinking, learning, memory and behavior of elderly people, that has become the hotspot nowadays for its increasing risks and incurability. The above fields have been intensively studied, and the link between the two has been demonstrated, whereas the way how GPCRs perturb AD progress are yet to be further explored given their complexities. In this review, we summarized recent progress regarding the GPCRs interacted with β-site APP cleaving enzyme 1 (BACE1), a key secretase in AD pathogenesis. Then we discussed the current findings on the regulatory roles of GPCRs on BACE1, and the possibility for pharmaceutical treatment of AD patients by the allosteric modulators and biased ligands of GPCRs. We hope this review can provide new insights into the understanding of mechanistic link between GPCRs and BACE1, and highlight the potential of GPCRs as therapeutic target for AD.

## Introduction

G protein coupled receptors (GPCRs) are integral membrane proteins that are used by cells to convert extracellular signals into intracellular responses, including responses to hormones, neurotransmitters, as well as responses to vision, olfaction and taste signals. These receptors form a superfamily of membrane proteins comprising of five distinct families on the basis of their sequences and structural similarities: rhodopsin (family A), secretin (family B), glutamate (family C), adhesion and Frizzled/Taste2 (Fredriksson et al., 2003; Rosenbaum et al., 2009). They all share common structural motifs in which seven transmembrane (TM) helices are connected to three extracellular loops and three intracellular loops. However, despite structural similarities, GPCRs have unique combinations of signal-transduction activities involving G protein dependent signaling pathways, as well as G protein-independent signaling pathways and complicated regulatory processes (Azzi et al., 2003; Rosenbaum et al., 2009; Rajagopal et al., 2010).

Numerous studies have presented evidence that implicate GPCRs in the pathogenesis of Alzheimer’s disease (AD) and in multiple stages of the hydrolytic processing of amyloid protein precursor (APP), a precursor protein involved in the formation of amyloid plaques found in AD patients’ brain (Thathiah and De Strooper, 2009, 2011; Wisely et al., 2014). Indeed accumulated data have shown that GPCRs can bind to β-secretase (β-site APP cleaving enzyme 1, BACE1) and γ-secretase which are key enzymes in the hydrolytic processing of APP (Liu et al., 2013; Nelson and Sheng, 2013; Thathiah et al., 2013). However there is currently no cure for AD and the hope for a new treatment has fallen short as the hottest inhibitors targeting BACE1 and γ-secretase haven’t been approved by the FDA due to the fact that they are not specific enough as they can also inhibit the normal biological functions of secretases. Therefore, the development of new therapeutic targets aimed at GPCRs could be a promising method to maintain the effect and control the side effects of inhibitors based on biased ligands or allosteric modulators (Rajagopal et al., 2010; Nickols and Conn, 2014; Violin et al., 2014).

The main purpose of this review is to provide an overview of the interaction between GPCRs and BACE1. We will firstly summarize the structures and signaling pathways of GPCRs, then address the reported implication of GPCRs in the pathologic process of AD, focusing on BACE1 related GPCRs, and also discuss the current findings on the regulatory roles of GPCRs in the pathological progression of AD, as well as the implication of GPCRs for pharmaceutical treatment of AD patients.

## A Profile of GPCRs

GPCRs are encoded by nearly 800 distinct genes in the human genome and form the largest TM receptor family found in humans (Bockaert and Pin, 1999). It has been estimated that more than half of all modern drugs are targeted at these receptors (Hopkins and Groom, 2002). Nevertheless, these developed drugs only target a very small number of GPCRs, leaving an enormous potential for drug developments within this field (Fredriksson et al., 2003).

**Figure 1:**
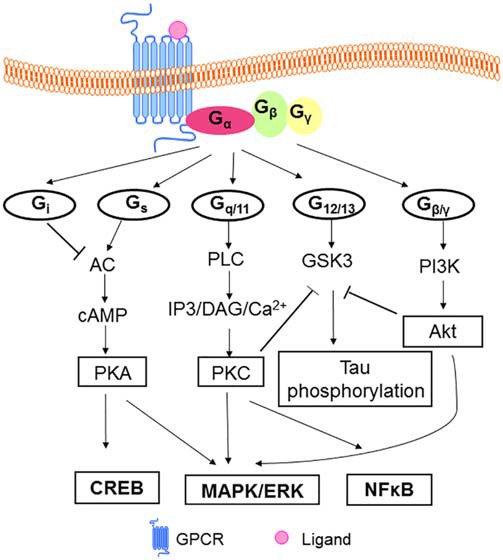
**Model of GPCRs mediated signaling pathways.** In classical model, heterotrimeric G proteins (α, β, γ subunits) mediate signal transduction via the receptor. Signal transduction initiated when ligands bind to GPCRs. The resulting conformation change promotes the exchange of GDP for GTP on the Galpha subunit of G proteins. G_s_ activates AC leading to the production of cAMP in cells, then cAMP binds to the regulatory subunit of PKA, regulating the phosphorylation of the GPCR and leading the process of desensitization of GPCR. PKA can regulate the level of CREB (Zeitlin et al., 2011) and mediates MAPK ERK pathway (New and Wong, 2007). G_q/11_ controls the activity of PLC, which hydrolyzes PIP2 to generate IP3 and DAG. IP3 and DAG in turn lead to an increase in the intracellular concentrations of free Ca^2+^, regulation of NFκB (Arendash et al., 2009) and the activation of a number of protein kinases and pathways, including PKC/MAPK/ERK (Ritter and Hall, 2009). GPCR activate PI3K/Akt cascades through G_βγ_ (New and Wong, 2007). Gi inhibits AC, and G_12/13_ is suggested to activate GSK3 in neuronal cells (Sayas et al., 2002a,b). GSK3 is involved in tau phosphorylation with regard to AD pathology (Ly et al., 2013). And Akt and PKC can inhibit GSK3 activity (New and Wong, 2007; Langmead et al., 2008). Abbreviations: GPCR(s), G protein-coupled receptor(s); GDP, guanosine diphosphate; GTP, guanosine triphosphate; AC, adenyl cyclase; cAMP, cyclic adenosine monophosphate; PKA, protein kinase A; CREB, cAMP response element-binding protein; MAPK, mitogen-activated protein kinase; ERK, extracellular signal-regulated kinase; PLC, phospholipase C; PIP2, phosphatidylinositol 4, 5-bisphosphate; IP3, inositol triphosphate; DAG, diacylglycerol; NFκB, nuclear factor kappa-light-chain-enhancer of activated B cells; PKC, protein kinase C; PI3K, phosphotidylinositol 3-kinase; Akt, protein kinase B; GSK3, glycogen synthase kinase-3; AD, Alzheimer’s Disease.

A GPCR is basically composed of three parts: the extracellular region, the TM region, and the intracellular region. The extracellular region contains N terminus and three extracellular loops (ECL1–ECL3); the TM region contains seven TM α-helices (TM1–TM7); the intracellular region contains three intracellular loops (ICL1–ICL3) and an intracellular amphipathic short α-helix (H8) lying perpendicular to the membrane plane, and the C terminus (Venkatakrishnan et al., 2013). In a broad sense, the extracellular region modulates ligand access, the types of which can vary tremendously, ranging from small molecules to large proteins. GPCRs can transduct signals received from messengers such as ions, organic odorants, amines, peptides, proteins, lipids, nucleotides, and even photons (Rosenbaum et al., 2009); the TM region forms the structural core, binds to ligands and transduces this information to the intracellular region through conformational changes, and the intracellular region interfaces with cytosolic signaling proteins. The main feature of GPCRs is to interact with G proteins. GPCRs can bind to different isoforms of G proteins: G_s_, G_q/11_, G_i_, G_12/13_ (Ferguson, 2007; Ritter and Hall, 2009) and activate a number of alternative signaling cascades inside cells, enabling functional diversities (Fredriksson et al., 2003). For details of GPCR-dependent signaling pathways see Figure 1. The predominant signaling pathway have been revealed from early studies of agonist-activated human β_2_ adrenergic receptor (β_2_AR) binding to G_s_, the stimulatory G protein for adenylyl cyclase. However, it is now known that the β_2_AR couples to the G protein G_i_ (Daaka et al., 1997), as well as activating G protein-independent pathways through β-arrestins (Azzi et al., 2003), a kind of scaffolding proteins mediating receptor desensitization and internalization (Rajagopal et al., 2010), and possibly other cellular signaling proteins. In addition, these activities are further complexified by factors such as GPCR oligomerization, localization to specific membrane compartments and resulting in differences in their lipid-bilayer composition (Rosenbaum et al., 2009). It was originally thought that most ligands bound to GPCRs have balanced or unbiased activities for signaling through β-arrestins and G protein pathways, however, some receptor-ligand systems and some allosteric modulators display biased signaling (see “Discussion” Section below), which have important implications for the design of therapeutics (Rajagopal et al., 2010).

To provide insights into the structural and functional diversity of GPCRs, the resolution of structure is necessary. However they have been proven to be difficult to obtain, typically requiring considerable time and investment (Venkatakrishnan et al., 2013). Indeed, GPCRs are naturally produced but only in very small quantities and often have short half-lives until degradation occurs in cells. Since GPCRs are membrane-bound proteins, indicating that they contain hydrophobic parts, thus experimental determination of their 3D structures is still an extremely difficult task (Warne et al., 2008). The first GPCR to yield molecular structure data was unliganded bovine rhodopsin purified from native tissue because of its high natural abundance in retina (Palczewski et al., 2000). We summarized the resolved GPCRs in Table 1. Data were collected from Protein Data Bank.

**Table 1:** **GPCRs that have resolved crystal structures in a timeline**.

Year	Proteins
2000	Bovine rhodopsin (1F88), Palczewski et al. (2000)
2007	Human **β**_2_AR (2RH1), Rasmussen et al. (2007)
2008	Turkey **β**_1_AR (2VT4), Warne et al. (2008); Squid rhodopsin (2Z73), Murakami and Kouyama (2008); Human A_2A_ AR (3EML), Jaakola et al. (2008)
2010	Human CXCR4 (3ODU), Wu et al. (2010); Human D3R (3PBL), Chien et al. (2010)
2011	Human H_1_R (3RZE), Shimamura et al. (2011)
2012	Human M_2_ AchR (3UON), Haga et al. (2012); Human S1PR (3V2Y), Hanson et al. (2012); Human M_3_ AChR (4DAJ), Kruse et al. (2012); Human KOR (4DJH), Wu et al. (2012); Mouse MOR (4DKL), Manglik et al. (2012); Human N/O FQR (4EA3), Thompson et al. (2012); Mouse DOR (4EJ4), Granier et al. (2012); Rat NTSR1 (4GRV), White et al. (2012); Human CXCR1 (2LNL), Park et al. (2012); Human PAR1 (3VW7), Zhang et al. (2012b)
2013	Human 5-HT_2B_R (4IB4), Wacker et al. (2013); Human 5-HT_1B_R (4IAR), Wang et al. (2013a); Human SMOR (4JKV), Wang et al. (2013b); Human CRFR1 (4K5Y), Hollenstein et al. (2013); Human GCGR (4L6R), Siu et al. (2013); Human CCR5 CR Tan et al. (2013)
2014	Human GluR1 (4OR2), Wu et al. (2014); Human P2YR12 (4NTJ), Zhang et al. (2014); Human GluR5 (4OO9), Doré et al. (2014); Human GPR40 (4PHU), Srivastava et al. (2014)
2015	Human OX2 (4S0V), Yin et al. (2015); Human DOR (4RW4), Fenalti et al. (2015); Human P2YR1 (4XNW), Zhang et al. (2015a); Human AT_1_R (4YAY), Zhang et al. (2015b)

*Abbreviations: GPCRs, G protein-coupled receptors; β_1_/β_2_AR, β_1_/β_2_ adrenergic receptor; A_2A_ AR, adenosine A_2A_ receptor; CXCR4, C-X-C chemokine receptor type 4; D_3_R, D_3_ dopamine receptor; H_1_R: histamine H_1_ receptor; M_2_/M_3_ AChR, M_2_/M_3_ muscarinic acetylcholine receptor; S1PR, sphingosine-1 phosphate receptor; K/M/DOR, kappa/mu/delta opioid receptor; N/O FQR, nociceptin/orphanin FQ receptor; NTSR1, neurotensin receptor type 1; CXCR1, CXC chemokine receptor 1; PAR1, protease-activated receptor; 5-HT_1B/2B_R, 5-hydroxytryptamine receptor type 1B/2B; SMOR, smoothened receptor; CRFR1, Corticotropin-releasing hormone receptor 1; GCGR, glucagon receptor; CCR5 CR, C-C chemokine receptor type 5; GluR1/5, glutamate receptor type 1/5; P2YR1/12, purinergic G protein-coupled receptor 1/12; GPR40, G protein-coupled receptor 40; OX_2_, Orexin receptor type 2; AT_1_R, Angiotensin II type 1 receptor*.

## GPCRs and AD

**Figure 2:**
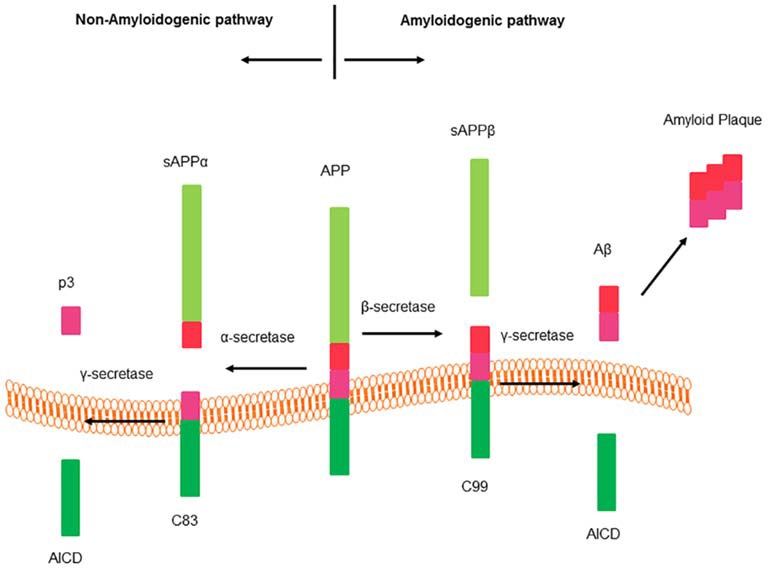
**Cleavage of amyloid precursor protein (APP).** APP processes two different hydrolysis pathways: amyloidogenic pathway and non-amyloidogenic pathway. Sequential cleavage of APP by α-secretase and γ-secretase generates a soluble amino terminal ectodomain of APP (sAPPα), the carboxy terminal fragment C83, APP intracellular domain (AICD) and a short fragment p3. There is no production of beta amyloid peptide (Aβ) from this pathway. Sequential cleavage of APP by β-secretase (BACE1) and γ-secretase generates sAPPβ, C99, AICD and Aβ (Thathiah and De Strooper, 2011).

AD is the most common neurodegenerative disorder afflicting around 24.0 million people worldwide, the morbidity rate of which rises dramatically as people get older (Erb et al., 2015). The pathological hallmark of AD is the extracellular deposition of beta amyloid peptide (Aβ), the oligomeric soluble forms of which are believed to be a key point for neuronal dysfunction, synapse loss, neurofibrillary degeneration (Das et al., 2016). Aβ comes from the amyloidogenic cleavage of APP, for details, see Figure 2.

Many reports presented evidence that GPCRs were related to AD (Blalock et al., 2004; Thathiah and De Strooper, 2009, 2011; Conn et al., 2014; Huang et al., 2015). Blalock et al. (2004) published that an alteration of the gene expression profile occurs in AD patients’ postmortem brains obtained by cDNA microarray analysis. Analysis of the gene expression profile of AD patients with different pathological severity vs. normal age-matched controls showed that the levels of transcripts from a number of GPCR genes changed, among which were inflammation associated GPCRs, hormone receptors, neurotransmitter receptors and some others. For example, arginine vasopressin receptor 1A, dopamine receptor D2 (D2R), metabotropic glutamate receptor type 6 (mGluR6), histamine H4 receptor, G protein-coupled receptor 2 (GPR2) and some others were upregulated; while cannabinoid receptor type 1, gamma-aminobutyric acid receptors, 5-hydroxytryptamine receptor 1E/2A, parathyroid hormone 2 receptor, orphan G protein-coupled receptor 22 (GPR22) and some others were downregulated (Blalock et al., 2004). Given that the change of expression levels of GPCRs would have an effect on the related biological processes, these observations suggested a potential role of GPCR in the pathological progression of AD, which requires further investigation (Blalock et al., 2004). Until now, numerous promising works have been done to connect GPCRs and AD pathology based on three hypotheses: the Cholinergic hypothesis, the Tau hypothesis and the Amyloid hypothesis (Thathiah and De Strooper, 2009, 2011; Wisely et al., 2014).

We summarize the GPCRs involved in AD in Table 2. Most GPCRs in family A are classified as binding small ligands within their TM core. Family B can bind mid-size peptide hormones with their deep and open V-shaped crevice. Family C is characterized by a large N-terminal domain which is the recognition site for ligands (Katritch et al., 2013). Besides, matching electrostatic properties among ligands and binding pocket allows the discrimination between ligands of a wide range of sizes. For example Kappa opioid receptor has highly acidic patches at the extracellular side, which most likely interacts with the basic C-terminus of dynorphin; in contrast, Mu opioid receptor lacks negative surface potential reflecting the uncharged nature of enkephalins (Manglik et al., 2012). Serine and threonine residues in the C-terminal region and a serine-rich sequence in ICL3 are potential sites for phosphorylation. A recent research verified that site mutation at ICL3 on the corticotrophin releasing factor receptor 1 (CRFR1) decreases the interaction between CRFR1 and γ-secretase, and blocks CRF-induced increase in total Aβ and Aβ40 but not Aβ42 (Park et al., 2015).

**Table 2:** **GPCRs involved in AD**.

GPCRs	Families	Relation to AD
M1 AChR	Family A	(sAPP↑, Aβ↓), Nitsch et al. (1992); Jones et al. (2008); and Jiang et al. (2012); (sAPP↑), Buxbaum et al. (1992); (BACE1↑), Züchner et al. (2004); (M1 AChR knock-out transgenic mice APP_Swedish/Indiana_, Aβ↑), Davis et al. (2010); (BACE1↓, Aβ↓), Caccamo et al. (2006)
M2 AChR	Family A	(BACE1↓), Züchner et al. (2004)
M3 AChR	Family A	(sAPP↑, Aβ↓), Nitsch et al. (1992); ( BACE1↑), Züchner et al. (2004)
mGluR1	Family C	(C83, C99, Aβ_40_↑), Kim et al. (2010); (sAPP↑), Lee et al. (1995); Kirazov et al. (1997) and Nitsch et al. (1997)
mGluR2	Family C	(tau phosphorylation↑), Lee et al. (2009); (C83, C99, Aβ_42_↑), Kim et al. (2010)
mGluR3	Family C	(C83, C99, Aβ_42_↑), Kim et al. (2010)
mGluR5	Family C	(C83, C99, Aβ_40_↑), Kim et al. (2010)
5-HT_2_R	Family A	(sAPP↑, Aβ↓), Nitsch et al. (1996); (Aβ↓), Arjona et al. (2002)
5-HT_4_R	Family A	(sAPPα↑, Aβ↓), Robert et al. (2001); Giannoni et al. (2013); Tesseur et al. (2013); and Pimenova et al. (2014)
5-HT_6_R	Family A	(Improved cognition and memory), Upton et al. (2008); Maher-Edwards et al. (2010); Rossé and Schaffhauser (2010); and Benhamú et al. (2014)
DOR	Family A	(Aβ↓), Teng et al. (2010) and Cai and Ratka (2012)
Adrenergic Receptor	Family A	(Aβ↑), Ni et al. (2006) and Chen et al. (2014); (tau phosphorylation↑), Branca et al. (2014) and Wisely et al. (2014)
ATR	Family A	(Tau phosphorylation and neurodegeneration↑), (AbdAlla et al., 2009a, b); (memory↓), Ongali et al. (2014)
Adenosine Receptor	Family A	(Aβ↓), Canas et al. (2009); Espinosa et al. (2013); Giunta et al. (2014); Nagpure and Bian (2014); and Orr et al. (2015); (BACE1↓, Aβ↓), Arendash et al. (2006)
CXCR2	Family A	(Aβ↑), Bakshi et al. (2008); Bakshi et al. (2009); Bakshi et al. (2011)
CXCR3	Family A	(plaque↑), Krauthausen et al. (2015)
CRFR1	Family B	(Aβ↓), Justice et al. (2015); (tau hyperphosphorylation↓), Carroll et al. (2011) and Rissman et al. (2012); (hippocampal synaptophysin level↑), Scullion et al. (2013)
PACR1	Family B	(sAPPα↑), Kojro et al. (2006); (cognition↑), Rat et al. (2011) and Yang et al. (2015)
GPR3	Family A	(Aβ↑), Thathiah et al. (2009) and Nelson and Sheng (2013)
P2Y receptor	Family A	(Aβ↑), Ajit et al. (2014) and Erb et al. (2015)
CX3CR1	Family A	(amyloid plaque↓), Lee et al. (2010); Liu et al. (2010); Cho et al. (2011) and Condello et al. (2015)
CCR2	Family A	(Aβ↓); El Khoury et al. (2007)

*Abbreviations: GPCRs, G protein-coupled receptors; AD, Alzheimer’s Disease; M_1_/ M_2_/M_3_ AChR, M_1_/ M_2_/M_3_ muscarinic acetylcholine receptor; mGluR1/2/3/5, metabotropic glutamate receptor type 1/2/3/5; 5-HT_2/4/6_R, 5-hydroxytryptamine receptor type 2/4/6; DOR: δ opioid receptor; ATR, angiotensin receptor; CXCR2/3, CXC chemokine receptor 2/3; CRFR1, corticotrophin releasing factor receptor 1; PACR1, Pituitary adenylate cyclase-activating polypeptide (PACAP) receptor type I; GPR3, G protein-coupled receptor 3; P2Y receptor, purinergic G protein-coupled receptor; CX3CR1, CX3C chemokine receptor type 1; CCR2, C-C chemokine receptor type 2; sAPP, soluble amino-terminal ectodomain of amyloid precursor protein; sAPPα, soluble amino terminal ectodomain of APP cleaved by α-secretase; Aβ, beta amyloid peptide; Aβ_40/42_, 40/42-amino acid beta amyloid peptide; BACE1, beta secretase; C83, 83-amino acid carboxy-terminal fragment; C99, 99-amino acid carboxy-terminal fragment*.

## GPCRs and BACE1

BACE1, a 501 amino acid type 1 TM aspartic protease related to the pepsin family, initiates Aβ generation and the resultant cerebral amyloidosis: deposition of Aβ (for details, see Figure 2). BACE1 catalytic domain contains two signature aspartic protease motifs (Asp-Thr/Ser-Gly-Ser/Thr) that form the active site of the enzyme and are oriented in the lumen of acidic intracellular compartments for cleaving the β-secretase site of APP. The highest concentrations of BACE1 can be found in neurons. With the correct sequence specificity and at acidic pH optimum for enzymatic activity, BACE1 undertakes processing of APP, and increases Aβ generation (Yan and Vassar, 2014). BACE1 is predominantly localized in the tans Golgi network (TGN) and endosomes. These acidic endosome compartments provide a low pH environment, which is more favorable for BACE1 activity (Das et al., 2016). In the AD brain, the activity of BACE1 has been shown to be up-regulated, but not its mRNA levels (Yang et al., 2003). Several proteins, such as translation initiation factor eIF2α (O’Connor et al., 2008), Golgi-localized γ-ear-containing adenosine diphosphate (ADP)-ribosylation factor binding proteins (GGAs; He et al., 2005), glycogen synthase kinase 3 (GSK3; Ly et al., 2013), the reticulon/Nogo family of proteins (He et al., 2004), and sortilins (Okada et al., 2010), are implicated in the regulation of BACE1, but the mechanisms, as well as their putative coordinated actions, remain unclear. In the GPCR superfamily, M1 AChR (Jiang et al., 2012), δ-opioid receptor (DOR; Teng et al., 2010), A_2A_ receptor (Arendash et al., 2006), are reported to regulate the activity of BACE1. GPCR regulating proteins, such as GPCR-associated sorting proteins (GASPs; Mishra and Heese, 2011), small G proteins such as Rabs (Teng et al., 2010; Buggia-Prévot et al., 2014) and ADP-ribosylation factor 6 (ARF6; Sannerud et al., 2011) are also revealed to mediate BACE1 activity.

### Muscarinic Acetylcholine Receptor

#### Introduction to Muscarinic Acetylcholine Receptor

The muscarinic acetylcholine receptors are members of the family A GPCRs that are synthesized by cholinergic cells. They are widely expressed in the central nervous system where they control a variety of neuronal functions (Langmead et al., 2008). The five subtypes of muscarinic acetylcholine receptor (mAChR) are generally divided into two groups based on signal transduction (Wess et al., 2007). M_1_, M_3_ and M_5_ mAChRs can activate phospholipase C (PLC) and mobilize intracellular calcium through G_q/11_, which is critical in neuronal communication and synaptic plasticity. While M_2_ and M_4_ mAChRs are coupled to G_i_ and then inhibit adenylate cyclase activity (Langmead et al., 2008) as well as several ion channels such as N-methyl-D-aspartate receptor (NMDAR; Salter and Kalia, 2004) and calcium channels (Zhou et al., 2008), leading to the reduction of cyclic adenosine monophosphate (cAMP), the inhibition of voltage-gated Ca^2+^ channels, and the increasing efflux of K^+^, in general, leading to inhibitory effects (Odagaki et al., 2014). mAChRs and ligand-gated ion channel nicotinic (nAChR) together can mediate the actions of acetylcholine (ACh). Both are important neurotransmitter receptors involved in learning and memory (Thathiah and De Strooper, 2009). Actually, commercial therapies for the treatment of AD approved by the FDA are mainly acetylcholinesterase inhibitors (AChEI) that are designed to boost levels of ACh. Since 1992, when researchers found that activation of M_1_ and M_3_ AChR can increase the release of soluble amino terminal ectodomain of APP cleaved by α-secretase (sAPPα). In addition, mAChRs have been intensively studied and revealed that the increase of sAPPα was accompanied by a decreased release of Aβ, suggesting that normal cholinergic activity may suppress the formation of potentially amyloidogenic derivatives (Nitsch et al., 1992). It was speculated that mAChR regulates APP release by protein kinase C (PKC) activation or an interaction of diacylglycerol and calcium released from internal pools by inositol triphosphate (IP3; Nitsch et al., 1992). The predominant mAChR in the CNS is subtype 1, which is located in the cerebral cortex and hippocampus, areas known to be vital for learning and memory and to be the location where amyloid plaques form, resulting in neuron loss. As a consequence, M1 mAChR agonists has been suggested as a promising novel approach to AD therapy (Langmead et al., 2008; Conn et al., 2009; Melancon et al., 2013). Xanomeline is a selective agonist of M_1_/M_4_ subtype, which provides the most significant human data for treatment of AD (Bodick et al., 1997). Despite the fact that it failed during phase-II clinical trial due to serious side-effects, Xanomeline has been shown to have reasonable efficacy to improve learning and short-term memory in AD patients (Conn et al., 2009).

#### BACE1 Related Muscarinic Acetylcholine Receptors

Studies have demonstrated that mAChR can mediate the level of BACE1 (Züchner et al., 2004; Jiang et al., 2012). The activation of M_1_ and M_3_ mAChRs in SK-SH-SY5Y neuroblastoma cell line by talsaclidine, a M_1_/M_3_-selective mAChR agonist, could up-regulate BACE1 in a dose-dependent way after 3 h treatment. In contrast, BACE1 expression was down-regulated by the activation of M_2_ mAChR (Züchner et al., 2004). However, results are controversial (Caccamo et al., 2006; Jiang et al., 2012). Caccamo et al. (2006) investigated the therapeutic efficacy of selective M_1_ mAChR agonist AF267B in the 3 × Tg-AD model mice (human APP Swedish mutation, APP_Swe_, tau_P30L_, mutant PS1_M146V_ knockin) and found a significant decrease in BACE1 levels in the brain of AF267B treated mice vs. untreated 3 × Tg-AD mice. Conversely, administration of dicyclomine, an M_1_ mAChR antagonist, led to a notable increase in BACE1 levels compared with phosphate-buffered saline (PBS)-injected mice (Caccamo et al., 2006). A recent data also showed a dramatical decrease of BACE1 protein levels instead of the mRNA levels after over-expression of M_1_ mAChR into human embryonic kidney (HEK)-APP_Swe_ cells, a HEK cell line stably expressing human APP Swedish mutations (Jiang et al., 2012). This process is accompanied with a rise of sAPPα and a fall of Aβ, while there is no effect on the level of full-length APP. The authors confirmed the interaction between M_1_ mAChR and BACE1 by yeast two-hybrid and co-immunoprecipitation experiments. They further silenced M_1_ mAChR and the endogenous BACE1 was markedly increased (Jiang et al., 2012).

### Opioid Receptor

#### Introduction to Opioid Receptor

The opioid system modulates several physiological processes, including analgesia, the stress response, the immune response and the neuroendocrine function. Opioid receptors and opioid peptides, vulnerable to AD, are widely expressed in the central nervous system, including hippocampus and cortex, the brain regions crucial for cognition. They play important roles in synaptic activation, learning and memory. Administration of opioid antagonists has been found to significantly improve the memory of animals (Gallagher et al., 1983); thus, in 1980s, the opioid antagonist naloxone, which was approved for the treatment of opioid overdose by the FDA in 1971, was used in a double-blinded placebo-controlled clinical study to test its potential effect on improving cognitive functioning in individuals with probable AD (Reisberg et al., 1983). However, later studies have failed to support the efficacy of the nonselective antagonists naloxone or naltrexone in AD (Tariot et al., 1986; Henderson et al., 1989). Given the distinct and even opposing roles of DOR, κ-opioid receptor (KOR), and μ-opioid receptor (MOR) in modulating animal behaviors, such as response of locomotion, level of anxiety, depressive-like behavior or alcohol intake in different opioid receptor knockout mice (Kieffer and Gavériaux-Ruff, 2002), the overlap between the distribution of opioid receptors and the location of amyloid plaques in AD patients led us to postulate a possible role of these three opioid receptors in the pathology of AD.

#### BACE1 Related Opioid Receptors

Previous reports have shown that altered cell signaling of opioid receptors is related to abnormal Aβ production and AD pathogenesis (Reisberg et al., 1983; Tariot et al., 1986; Henderson et al., 1989; Ni et al., 2006). Teng et al., (2010) over-expressed DOR in HEK293T cell line and performed the fluorogenic substrate assay to directly evaluate the effect of DOR on secretase activities and found that 30 min after stimulation by DOR agonist, BACE1 and γ-secretase activities were enhanced to 143% and 156%, respectively, while the activity of α-secretase was not affected. Pretreatment with DOR selective antagonist naltrindole (NTI), on the other hand, blocked the enhancement of BACE1 and γ-secretase activities by DOR agonist, indicating that secretase activity enhancement depends on DOR activation (Teng et al., 2010). Chronic treatment of APP/PS transgenic AD model mice with NTI alleviated Aβ pathology and improved cognitive deficits in spatial reference memory by reducing activities of endogenous BACE1 and γ-secretase without any changes in APP expression levels or Aβ clearance (Teng et al., 2010).

### Adenosine Receptor

#### Introduction to Adenosine Receptor

Adenosine is found in all cells including glia and neurons, and plays important roles in the regulation of synaptic transmission and neuronal excitability in the central nervous system. Functional and molecular studies made it possible to classify adenosine receptors as A_1_, A_2A_, A_2B_, and A_3_ subtypes (Ribeiro and Sebastião, 2010). A_1_ receptors are highly enriched in the CA1 region of hippocampus in a normal healthy brain. A change in the pattern of A_1_ receptor expression has been found in AD patients when compared with age-matched control brains (Angulo et al., 2003). In addition, activation of A_1_ receptors could lead to the production of soluble APP, which was confirmed by the use of A_1_-selective antagonist DPCPX (Angulo et al., 2003). Studies also revealed that A_1_ receptors mediate tau phosphorylation, another key factor for pathogenesis of AD besides Aβ, and its translocation towards the cytoskeleton of neuroblastoma cells (Angulo et al., 2003; Giunta et al., 2014). A marked increase in A_1_ receptor immunoreactivity has been found in degenerating neurons with neurofibrillary tangles and in dystrophic neurites of Aβ plaques in the hippocampus and frontal cortex of AD (Angulo et al., 2003). Significant co-localizations of A_1_ receptors and Aβ in senile plaques, as well as of A_1_ receptors and tau in neurons with tau deposition, have been found (Angulo et al., 2003; Giunta et al., 2014). The A_2A_ receptor is also expressed in the brain, where it has important roles in the regulation of glutamate and dopamine release, making it a potential therapeutic target for the treatment of neuronal diseases. A_2A_ receptors have low expression in healthy brain but this pattern of expression and functionality can be changed in pathological conditions.

#### BACE1 Related Adenosine Receptors

Arendash et al. reported a decrease of BACE1 and PS1 expression level in APP transgenic mouse hippocampal tissue after long term administration of caffeine, a non-selective A_1_ and A_2A_ adenosine receptor antagonist, to APP_Swe_ transgenic mice. Caffeine treatment also improved cognition and reduced Aβ_40_ and Aβ_42_ generation of APP_Swe_ mice (Arendash et al., 2006). The authors then further provided evidence that caffeine treatment can reverse cognitive impairment and they developed insight into possible mechanisms involved in BACE1 suppression by caffeine (Arendash et al., 2009; Zeitlin et al., 2011).

### GPCR Regulating Proteins

#### Small GTPase

Small GTPase constitute a superfamily consisting of more than 100 members. This superfamily is structurally classified into at least five families: the Ras, Rho/Rac/Cdc42, Rab, Sar1/ARF, and Ran families (Takai et al., 2001). Some Rabs are expressed ubiquitously in human tissues, whereas others are tissue-specific. Within cells, they are localized to the cytosolic face of distinct intracellular membranes (Pfeffer, 2013; Nagano et al., 2015). Functional loss of the Rab pathways has been implicated in a variety of diseases, Rab5 and Rab7, which control early and late endosome fusion respectively, are selectively up-regulated in hippocampal neurons of individuals with mild cognitive impairment and AD (Ginsberg et al., 2010). In the team of Pei G, they observed the colocalization of Rab7 and BACE1, Rab7 and BACE1/PS1 complex (Teng et al., 2010). Further data from RNAi screening of all human Rab-GTPase associate Rab11 with late onset AD (Udayar et al., 2013). Recently, Buggia et al. reported that Rab11 is critical for axonal sorting of BACE1, since BACE1 shows in Rab11 positive endosomes, and impairment of Rab11 function leads to a diminution of total and endocytosis BACE1 in axons (Buggia-Prévot et al., 2014). Rab11 was revealed to colocalize with BACE1 by direct visual images (Das et al., 2016). Also ARFs are a family of small GTPases that are involved in various aspects of membrane trafficking events. ARF6 is demonstrated to mediate the endosomal sorting of BACE1. Furthermore, the sorting of newly internalized BACE1 from ARF6-positive towards Rab GTPase 5 (Rab5)-positive early endosomes depends on the carboxyterminal short acidic cluster-dileucine motif of BACE1 (Sannerud et al., 2011). Ras homolog enriched in brain (Rheb) was demonstrated to regulate BACE1 stability and activity by reducing the half-life of BACE1 in a GTP-dependent manner (Shahani et al., 2014).

#### GPCR-Associated Sorting Protein

GPCR-associated sorting protein (GPRASP family protein) has been shown to preferentially sort a number of native GPCRs to the lysosome for degradation after endocytosis. p60TRP, also known as GASP3 or BHLHB9, is a kind of GPRASP. P60TRP is localized in both the cytoplasm and the nucleus of cells and has been predominantly observed in the CNS, particularly in the brain. Among the many distinguishing features of p60TRP, one of the most noteworthy is that it contains a potential myc-type basic helix-loop-helix (bHLH) domain at its C-terminus; this domain is a protein structural motif that characterizes a family of transcription factors (Heese, 2013). Since 2004, Heese Klaus with the lab members have discovered p60TRP gene as a potential rescue factor against cell death by applying a death trap method (Heese et al., 2004). Increased expression of p60TRP induces the dephosphorylation of APP, which inhibits BACE1 activity and causes reduced APP intracellular domain (AICD) signaling in p60TRP-overexpressing cells (Mishra and Heese, 2011).

## Discussion

How do GPCRs regulate the levels of BACE1? Despite countless research studies being performed, the answer remains to be elucidated. However, the available documents provide us insights to the role of GPCRs in mediating BACE1 by mechanisms that fall into the following categories: (i) GPCRs activation stimulates G protein-dependent signaling pathway, which finally alters the expression level of BACE1; (ii) GPCRs activation mediates the degradation of BACE1; and (iii) GPCRs activation regulates the trafficking of BACE1.

### Impact of GPCRs Signaling Pathway on BACE1 Expression

The classical G protein signaling pathway (see Figure 1) is considered to be an explanation for the regulation of BACE1 and other key proteins in AD (Thathiah and De Strooper, 2011). The concurrence of changes in some molecules in the pathway and changes in key proteins in AD after activation of GPCRs seems to be more than coincidental.

A previous study suggested that the mitogen-activated protein kinase (MAPK)/extracellular signal-regulated kinase (ERK) pathway is involved in the regulation of BACE1 by M_1_ mAChR (Sinha et al., 1999). One effect of MAPK/ERK activation is to alter the translation of mRNA to proteins. Züchner et al. showed that agonists binding to M_1_-/M_3_- mAChR can up-regulate BACE1 expression through activation of both PKC and MAPK signaling cascades. In contrast, studies have shown BACE1 expression is down-regulated by the activation of M_2_- mAChR- and protein kinase A (PKA)-mediated pathways (Züchner et al., 2004). Studies also showed that caffeine treatment can significantly increase PKA activity in Tg mice and striatum of APP_Swe_ mice (Arendash et al., 2009; Zeitlin et al., 2011). The author proposed that enhanced PKA activity inhibits cRaf-1 (a proto-oncogene serine/threonine-protein kinase) by phosphorylation at serine259, decreasing nuclear factor kappa-light-chain-enhancer of activated B cells (NFκB) activity and the expression of NFκB-controlled genes such as BACE1 in the hippocampus of treated AD Tg mice (Arendash et al., 2009). In addition, the results showed that caffeine increases cAMP response element-binding protein (CREB) phosphorylation at Ser133 in the striatum and decreases c-Jun N-terminal kinase (JNK)/ERK phosphorylation in the striatum and cortex of APP_Swe_ mice (Zeitlin et al., 2011). And Caccamo et al. (2006) demonstrated a significant decrease in BACE1 levels in the brain of AF267B treated mice and compared to untreated 3 × Tg-AD mice, while a dramatic increase in BACE1 levels in the brain of dicyclomine treated mice. Accompanied with the changes of BACE1, a marked rise of phosphorylated ERK in the brains of AF267B treated mice, and a significant reduction of that in the brains of dicyclomine treated mice, in comparison with PBS-treated mice, while levels of ERK were not changed in the brain of AF267B or dicyclomine treated mice (Caccamo et al., 2006). This is consistent with the fact that phosphorylated ERK represents functional ERK. Paradoxically, recent data suggested M_1_ mAChRs can interact with BACE1 and decrease BACE1 to reduce Aβ, in which process ERK and phosphoinositide 3-kinase (PI3K) signaling pathway is not involved (Jiang et al., 2012). Also, G protein pathway is not involved in the DOR activation-induced rise of BACE1 (Teng et al., 2010).

Furthermore, comparable findings were observed regarding γ-secretase and GPCRs (Ni et al., 2006; Thathiah et al., 2009). Pei G and his team ruled out the involvement of β_2_-adrenergic receptor (β_2_-AR)-induced G protein-dependent signaling pathway. Initially, they used β_2_-AR mutants, which could not activate G protein, and found that the uncoupling of the receptors with G protein did not affect the enhancement of γ-secretase. Then they treated cells with some reagents that mimic G protein activation and found none of them could enhance γ-secretase activity (Ni et al., 2006). Thathiah et al. (2009) demonstrated that the orphan G protein-coupled receptor 3 (GPR3) could increase Aβ generation by enhancing γ-secretase activity independent of GPCR signaling pathway with similar methods. GPR3, G protein-coupled receptor 6 (GPR6) and G protein-coupled receptor 12 (GPR12) shared signaling triggered by sphingosine-1 phosphate receptor (S1PR; Uhlenbrock et al., 2002). They are involved in cAMP signaling pathway (Hinckley et al., 2005; Tanaka et al., 2007).

### Impact of GPCRs on BACE1 Degradation

BACE1 has been reported to be degraded by the lysosomes and ubiquitin-proteasome pathway (Qing et al., 2004; Koh et al., 2005; Kang et al., 2012; Wang et al., 2012), and accelerating BACE1 degradation by ubiquitin carboxyl-terminal hydrolase L1 (UCHL1) reduces 99-amino acid carboxy-terminal fragment (C99) and Aβ production (Zhang et al., 2012a). Researchers from a lab of Xiamen University observed the increase and decrease of BACE1 after activation and inhibition of M_1_ mAChR, meanwhile the mRNA levels of BACE1 remained stable. They suggested that the effects of BACE1 by M_1_ mAChR is probably mediated by its degradation (Jiang et al., 2012). When cells were treated with proteasome inhibitor lactacystin, it was shown that over-expression of M_1_ mAChR could result in a marked increase in the level of ubiquitinated BACE1. When cells were treated with a lysosome inhibitor NH_4_Cl, it was found that BACE1 levels also increases, but no differences between cells of M_1_ mAChR over-expression treatment, suggesting that BACE1 could be degraded by both lysosome and ubiquitin-proteasome pathway, while M1 mAChR-mediated BACE1 degradation is mainly through ubiquitin-proteasome pathway (Jiang et al., 2012). Whereas the details about how GPCRs regulate BACE1 degradation needs further investigation.

### Impact of GPCRs on BACE1 Trafficking

BACE1 cycles between the Golgi apparatus and the plasma membrane, traveling through endosomes on the way. Substantive evidence indicated that APP processing by BACE1 is dependent on the intracellular trafficking of this enzyme (He et al., 2004; Tesco et al., 2007; Sannerud et al., 2011; Chia et al., 2013; Buggia-Prévot et al., 2014). For example, BACE1 can interact with reticulon/Nogo proteins, whose increased expression can block BACE1 in the endoplasmic reticulum (ER) that has a neutral pH environment and thus inhibiting BACE1 activity in Aβ generation (He et al., 2004; Murayama et al., 2006). Depletion of GGA proteins increases the accumulation of BACE1 in acidic early endosomes resulting in enhanced BACE1 stability and APP cleavage (Tesco et al., 2007). Nonetheless, knowledge of the intracellular trafficking pathway of internalized BACE1 remains in doubt.

#### Alteration of BACE1 in Plasma Membrane

Many GPCRs, BACE1, γ-secretase, and Aβ generation are localized in lipid raft (Thathiah et al., 2009; Teng et al., 2010; Park et al., 2015). Researches have revealed that GPCRs activation could increase the distribution of BACE1 and γ-secretase in lipid rafts (Teng et al., 2010). What’s more, they proposed a model of GPCR/BACE1/γ-secretase complex, based on the results of immunoprecipitation experiments. They then verified that disruption of lipid raft by removing cholesterol from the cells could significantly reduce the interaction between DOR and BACE1 or γ-secretase, indicating that the association is dependent on the integrity of lipid raft (Teng et al., 2010). Likewise, GPR3 appears to promote the trafficking of γ-secretase to the cell surface and increased localization in detergent-resistant membranes (DRMs), which eventually leads to an increase in Aβ generation (Thathiah et al., 2009).

#### Alteration of BACE1 Internalization

Clathrin-mediated endocytosis is the primary process of GPCRs and APP internalization, while internalization of BACE1 occurs at a slower bulk flow rate (Ni et al., 2006; Sannerud et al., 2011). Pei G and his group has confirmed that DOR activation leads to an enrichment of BACE1 and γ-secretase in endocytic compartments. They suspected whether DOR could direct the endocytosis of secretases, so they performed confocal fluorescence time-lapse microscopy with HEK293T cells expressing fluorescently tagged BACE1 and PS1 (catalytic subunit of γ-secretase) together with tagged DOR, they indeed detected a strong colocalization of the three proteins (Teng et al., 2010).

#### Alteration of BACE1 in Endosomes

Mechanistic studies have revealed that activated DOR can facilitate the endocytic sorting of secretases for APP endoproteolysis and enhance Aβ production. A dysfunction of the receptor can retard the endocytosis of BACE1 and γ-secretase and thus the production of Aβ (Teng et al., 2010). The authors further conceived two chimeric receptors to test whether different receptor endocytic sorting could coordinate with BACE1 and γ-secretase intracellular trafficking. Results indicated that different receptors regulate specific substrates (Teng et al., 2010).

**Figure 3:**
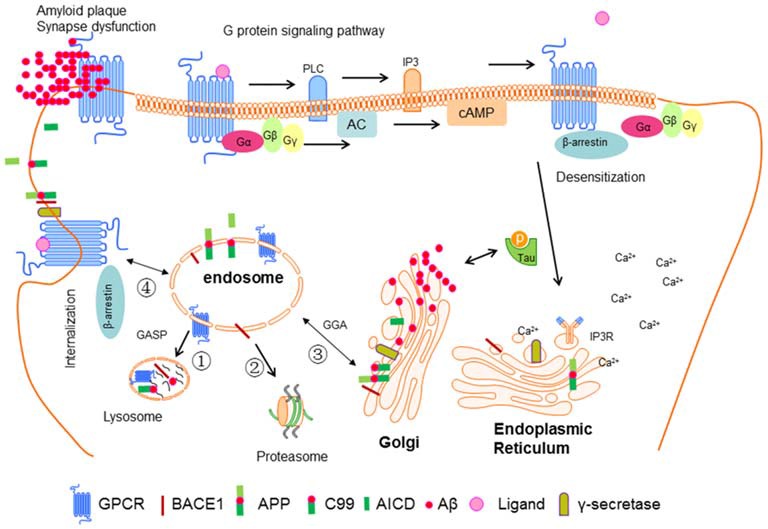
**Interaction between GPCR and BACE1 in neuronal cell.** Ligand-bind GPCR can activate G proteins and then stimulate downstream signal molecules such as PLC, IP3, AC, and cAMP. IP3 can bind to IP3 receptor in the endoplasmic reticulum (ER) leading the release of Ca^2+^. β-Arrestin can be recruited to GPCR mediating its desensitization, internalization and G protein independent signaling pathway. GPCR, BACE1, APP, and γ-secretase are synthesized in ER, and then be transported into plasma membrane, then be guided to endosome. After that these proteins can be sorted to lysosome, proteasome, ER, or back to plasma membrane. After sequential cut by BACE1 and γ-secretase, APP releases Aβ, which can form oligomers and fibers and finally senile plaque. In addition, soluble Aβ can promote the phosphorylation of tau, which in turn accelerates the aggregation of Aβ. These factors eventually cause the synapse dysfunction and AD.

The shedding of APP by BACE1 appears to mainly occur in early endosomal compartments based on the colocalization with Rab5, a marker for early endosomes. In this case, the factors promoting APP or BACE1 internalization to endosomes can enhance Aβ generation. Reversely, blocking their meeting decreases Aβ levels (Sannerud et al., 2011). The small GTPase ARF6 was reported to be an important modulator of BACE1 sorting. The authors created an ARF6 mutant locked in its ADP-bound state to evaluate the effect on BACE1 and APP sorting, and found that it blocks the delivery of BACE1 to early endosomes, suggesting that the GTP hydrolysis of ARF6 is required for the sorting of BACE1 (Sannerud et al., 2011). They further confirmed that the dileucine motif in the BACE1 carboxyterminal tail is required for the sorting of BACE1 to early endosomes (Sannerud et al., 2011). While further studies showed that the recycling endosomes marker Rab11 is colocalized with internalized BACE1, and the impairment of Rab11 activity caused accumulation of internalized BACE1 in the soma with a concomitant decrease of its expression levels in axons (Buggia-Prévot et al., 2014). The newest study revealed that recycling endosomes are the major locale of APP and BACE1 convergence in the dendrites by direct optical assay based on fluorescence complementation, and in the soma, APP and BACE1 interaction at the TGN (Das et al., 2016). In summary, the mechanisms are not clear yet. We draw a profile from the discussion above to provide a better reference for others (details see Figure 3).

## Perspectives

BACE1 has broad biological functions in cells, but in the respect of AD therapy development, scientists are devoted to searching a way of inhibiting its undue cleavage of APP without impacting other substrates that have important physiological functions *in vivo* (Vassar, 2014). Likewise, the most severe side effects due to absence of γ-secretase activity are caused by deficient Notch signaling (Selkoe and Kopan, 2003). Researchers found that GPR3 apparently affects the processing of APP, but not of Notch, which suggests that GPR3 is an interesting AD therapeutic target (Thathiah et al., 2009). However, clinical trials carried out so far have highlighted the difficulties involved in this type of anti-AD therapy. As evidenced by side effects, likely due to the ubiquitous nature of the secretases, it might cleave multiple substrates. Thus, combining potency, selectivity and the desired safety profiles remains to be a continued challenge. Hence, there is still a clear need for a novel biochemical research for the development of potent and selective modulators targeting BACE1 with properties optimal for central nervous system therapeutics (Yan and Vassar, 2014). The biased ligands and allosteric modulators of GPCRs provide the promising options (Khoury et al., 2014; Violin et al., 2014; Luttrell et al., 2015).

### Biased Ligands

**Figure 4:**
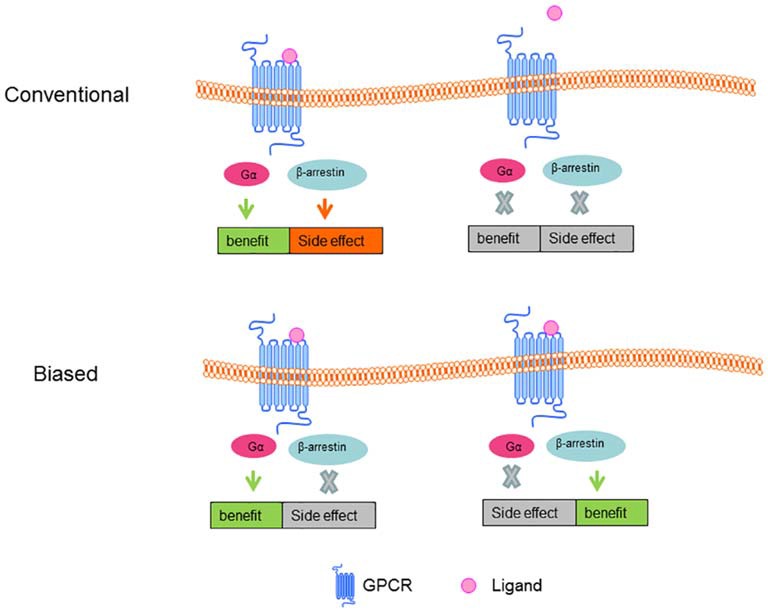
**Conventional and Biased ligand-GPCR signal pathway.** In conventional ligand-GPCR signal model, the benefit and side effect are thought to turn on or off at the same time; in biased ligand-GPCR signal model, benefit can be maintained while side effect can be eradicated.

Typically, GPCR activation is involved in broad networks of signaling pathways, in which most are mediated by G proteins and β-arrestins (Lefkowitz and Shenoy, 2005). Standard agonists and antagonists are able to activate or inactivate the entirety of a receptor’s signaling network. However, biased ligands (see Figure 4) can selectively engage some signals while avoiding other signaling pathways mediated by the same receptor (Wei et al., 2003). Biased ligands provide functional selectivity for pharmacology and gain increasing prominence. A handful of biased ligands that are specifically targeting G protein or β-arrestin signaling pathways have been discovered (Violin et al., 2014), among which ligands targeting angiotensin II type 1 receptor, β_2_AR, opioid receptors are the hottest. An example is applying biased agonist pilocarpine in the treatment of AD. Pilocarpine shows positive therapeutic effects in different AD models, specially, pilocarpine biased G_q_ mediates PLC activation over G_s_ medicated AC stimulation, whereas non-selective muscarinic agonist carbachol could equally stimulate G_s_ and G_q_ signaling pathways (Fisher et al., 1993).

### Allosteric Modulators

Conventional ligands belong to orthostatic GPCR agonists, and their cellular effects are mediated through interacting with the ligand-binding pocket and changing the distribution of conformation within the receptor ensemble. Allosteric modulators include ions, ligands, small and large molecules and protein complexes that modulate the coupling of receptors to their effectors by binding to GPCRs at a site other than the protein’s active sites, which are endogenous ligand binding sites. The conformational change caused by the allosteric ligands can selectively alter the reactivity of the receptor towards certain aspects of the pharmacology of the orthosteric ligand but not others, which engendered stimulus-bias. According to the effects on receptor signaling, allosteric ligands have been divided into two main categories: positive allosteric modulators (PAMs) and negative allosteric modulators (NAMs; Khoury et al., 2014). Two drugs have been launched: Sensipar/Mimpra cinacalcet (Amgen/NPS) is a PAM of calcium-sensing receptor (CaSR) for hyperparathyroidism treatment; Selzentry/Celsentri maraviroc (Pfizer) is a NAM of C-C chemokine receptor type 5 (CCR5) for human immunodeficiency virus (HIV) therapy. Much work has been done to screen the biased allosteric modulators for treatment of AD, among which, modulators targeting mAChRs lead the research. MK-7622 from Merck is a PAM of M_1_ receptor that is currently tested in phase II (Conn et al., 2014). The M_1_ AChR agonist AF267B can alleviate pathology of AD by shifting the processing of APP towards the non-amyloidogenic pathway (Caccamo et al., 2006). TBPB, a systemically M_1_ AChR allosteric agonist, was shown to decrease the level of APPβ (Jones et al., 2008). Data from early optimization and animal studies are indicative of a healthy pipeline of novel allosteric modulators that hold promise for the treatment of AD (Caccamo et al., 2006; Jones et al., 2008; Conn et al., 2014).

## Concluding Remarks

Collectively, AD is a complex disease and regarding the reported factors, one solution might not be enough to deal with the complex condition *in vivo* (Thathiah and De Strooper, 2009). It is clear that several GPCRs and GPCR related proteins are involved in the regulation of BACE1 and the pathogenesis of AD (Table 2). Nevertheless, progress in this field is hampered by the difficulties in screening ligands with high specificity and selectivity and by the adverse side effects of drugs in the pipeline. Studies have demonstrated that BACE1 and γ-secretase have other necessary functions in cells, simple segregation of these secretases will cause a hamper of their ordinary functions and lead to undesirable side effects, which are so overwhelming that they prohibit drug’s efficacy and approval. There is an urgency to understand mechanisms involved in how GPCRs mediate secretases in AD. According to the literature, GPCRs might regulate the secretases via G protein-dependent signaling pathways, or influence the degradation and internalization of secretases. However, this area is still filled with unsolved problems that need to be determined by further investigations. With the development of biased ligands and allosteric modulators of GPCRs, the therapeutic potential of GPCRs might be enhanced to provide alternative strategies for effectively modulating AD pathogenesis with fewer side effects in the future.

## Author Contributions

The review is conceived and designed by JZ and HQ, drafted and revised by JZ, HQ and YD, discussed by JZ, HQ and ZJ.

## Funding

This work is supported by National Natural Science Foundation of China under grant no. 8157051211.

## Conflict of Interest Statement

The authors declare that the research was conducted in the absence of any commercial or financial relationships that could be construed as a potential conflict of interest. The reviewer VEM and handling Editor declared a current collaboration and the handling Editor states that the process nevertheless met the standards of a fair and objective review.

## References

[B1] AbdAlla S. Lother H. el Missiry A. Langer A. Sergeev P. el Faramawy Y.. (2009a). Angiotensin II AT2 receptor oligomers mediate G-protein dysfunction in an animal model of Alzheimer disease. J. Biol. Chem. 284, 6554–6565. 10.1074/jbc.M807746200 19074441

[B2] AbdAlla S. Lother H. el Missiry A. Sergeev P. Langer A. el Faramawy Y.. (2009b). Dominant negative AT(2) receptor oligomers induce G-protein arrest and symptoms of neurodegeneration. J. Biol. Chem. 284, 6566–6574. 10.1074/jbc.m808277200 19074439

[B3] Ajit D. Woods L. Camden J. Thebeau C. El-Sayed F. Greeson G.. (2014). Loss of P2Y2 nucleotide receptors enhances early pathology in the TgCRND8 mouse model of Alzheimer’s disease. Mol. Neurobiol. 49, 1031–1042. 10.1007/s12035-013-8577-5 24193664 PMC3954904

[B4] Angulo E. Casadó V. Mallol J. Canela E. I. Viñals F. Ferrer I.. (2003). A1 adenosine receptors accumulate in neurodegenerative structures in alzheimer disease and mediate both amyloid precursor protein processing and tau phosphorylation and translocation. Brain Pathol. 13, 440–451. 10.1111/j.1750-3639.2003.tb00475.x 14655750 PMC8095992

[B5] Arendash G. W. Mori T. Cao C. Mamcarz M. Runfeldt M. Dickson A.. (2009). Caffeine reverses cognitive impairment and decreases brain amyloid-β levels in aged Alzheimer’s disease mice. J. Alzheimers Dis. 17, 661–680. 10.3233/JAD-2009-1087 19581722

[B6] Arendash G. W. Schleif W. Rezai-Zadeh K. Jackson E. K. Zacharia L. C. Cracchiolo J. R.. (2006). Caffeine protects Alzheimer’s mice against cognitive impairment and reduces brain β-amyloid production. Neuroscience 142, 941–952. 10.1016/j.neuroscience.2006.07.021 16938404

[B7] Arjona A. A. Pooler A. M. Lee R. K. Wurtman R. J. (2002). Effect of a 5-HT(2C) serotonin agonist, dexnorfenfluramine, on amyloid precursor protein metabolism in guinea pigs. Brain Res. 951, 135–140. 10.1016/s0006-8993(02)03153-0 12231467

[B8] Azzi M. Charest P. G. Angers S. Rousseau G. Kohout T. Bouvier M.. (2003). β-Arrestin-mediated activation of MAPK by inverse agonists reveals distinct active conformations for G protein-coupled receptors. Proc. Natl. Acad. Sci. U S A 100, 11406–11411. 10.1073/pnas.1936664100 13679574 PMC208770

[B9] Bakshi P. Jin C. Broutin P. Berhane B. Reed J. Mullan M. (2009). Structural optimization of a CXCR2-directed antagonist that indirectly inhibits γ-secretase and reduces Aβ. Bioorg. Med. Chem. 17, 8102–8112. 10.1016/j.bmc.2009.09.051 19853461

[B11] Bakshi P. Margenthaler E. Laporte V. Crawford F. Mullan M. (2008). Novel role of CXCR2 in regulation of γ-secretase activity. ACS Chem. Biol. 3, 777–789. 10.1021/cb800167a 19067586

[B10] Bakshi P. Margenthaler E. Reed J. Crawford F. Mullan M. (2011). Depletion of CXCR2 inhibits γ-secretase activity and amyloid-β production in a murine model of Alzheimer’s disease. Cytokine 53, 163–169. 10.1016/j.cyto.2010.10.008 21084199

[B12] Benhamú B. Martín-Fontecha M. Vázquez-Villa H. Pardo L. López-Rodríguez M. L. (2014). Serotonin 5-HT6 receptor antagonists for the treatment of cognitive deficiency in Alzheimer’s disease. J. Med. Chem. 57, 7160–7181. 10.1021/jm5003952 24850589

[B13] Blalock E. M. Geddes J. W. Chen K. C. Porter N. M. Markesbery W. R. Landfield P. W. (2004). Incipient Alzheimer’s disease: microarray correlation analyses reveal major transcriptional and tumor suppressor responses. Proc. Natl. Acad. Sci. U S A 101, 2173–2178. 10.1073/pnas.0308512100 14769913 PMC357071

[B14] Bockaert J. Pin J. P. (1999). Molecular tinkering of G protein-coupled receptors: an evolutionary success. EMBO J. 18, 1723–1729. 10.1093/emboj/18.7.1723 10202136 PMC1171258

[B15] Bodick N. C. Offen W. W. Levey A. I. Cutler N. R. Gauthier S. G. Satlin A.. (1997). Effects of xanomeline, a selective muscarinic receptor agonist, on cognitive function and behavioral symptoms in Alzheimer disease. Arch. Neurol. 54, 465–473. 10.1001/archneur.1997.00550160091022 9109749

[B16] Branca C. Wisely E. V. Hartman L. K. Caccamo A. Oddo S. (2014). Administration of a selective β2 adrenergic receptor antagonist exacerbates neuropathology and cognitive deficits in a mouse model of Alzheimer’s disease. Neurobiol. Aging 35, 2726–2735. 10.1016/j.neurobiolaging.2014.06.011 25034342 PMC4252846

[B17] Buggia-Prévot V. Fernandez C. G. Riordan S. Vetrivel K. S. Roseman J. Waters J.. (2014). Axonal BACE1 dynamics and targeting in hippocampal neurons: a role for Rab11 GTPase. Mol. Neurodegener. 9:1. 10.1186/1750-1326-9-1 24386896 PMC4031619

[B18] Buxbaum J. D. Oishi M. Chen H. I. Pinkas-Kramarski R. Jaffe E. A. Gandy S. E.. (1992). Cholinergic agonists and interleukin 1 regulate processing and secretion of the Alzheimer β/A4 amyloid protein precursor. Proc. Natl. Acad. Sci. U S A 89, 10075–10078. 10.1073/pnas.89.21.10075 1359534 PMC50280

[B19] Caccamo A. Oddo S. Billings L. M. Green K. N. Martinez-Coria H. Fisher A.. (2006). M1 receptors play a central role in modulating AD-like pathology in transgenic mice. Neuron 49, 671–682. 10.1016/j.neuron.2006.01.020 16504943

[B20] Cai Z. Ratka A. (2012). Opioid system and Alzheimer’s disease. Neuromolecular Med. 14, 91–111. 10.1007/s12017-012-8180-3 22527793

[B21] Canas P. M. Porciúncula L. O. Cunha G. M. A. Silva C. G. Machado N. J. Oliveira J. M. A.. (2009). Adenosine A2A receptor blockade prevents synaptotoxicity and memory dysfunction caused by β-amyloid peptides via p38 mitogen-activated protein kinase pathway. J. Neurosci. 29, 14741–14751. 10.1523/JNEUROSCI.3728-09.2009 19940169 PMC6665997

[B22] Carroll J. C. Iba M. Bangasser D. A. Valentino R. J. James M. J. Brunden K. R.. (2011). Chronic stress exacerbates tau pathology, neurodegeneration and cognitive performance through a corticotropin-releasing factor receptor-dependent mechanism in a transgenic mouse model of tauopathy. J. Neurosci. 31, 14436–14449. 10.1523/JNEUROSCI.3836-11.2011 21976528 PMC3230070

[B23] Chen Y. J. Peng Y. Che P. L. Gannon M. Liu Y. Li L.. (2014). α(2A) adrenergic receptor promotes amyloidogenesis through disrupting APP-SorLA interaction. Proc. Natl. Acad. Sci. U S A 111, 17296–17301. 10.1073/pnas.1409513111 25404298 PMC4260556

[B24] Chia P. Z. Toh W. H. Sharples R. Gasnereau I. Hill A. F. Gleeson P. A. (2013). Intracellular itinerary of internalised β-secretase, BACE1 and its potential impact on β-amyloid peptide biogenesis. Traffic 14, 997–1013. 10.1111/tra.12088 23773724

[B25] Chien E. Y. T. Liu W. Zhao Q. A. Katritch V. Han G. W. Hanson M. A.. (2010). Structure of the human dopamine D3 receptor in complex with a D2/D3 selective antagonist. Science 330, 1091–1095. 10.1126/science.1197410 21097933 PMC3058422

[B26] Cho S.-H. Sun B. Zhou Y. Kauppinen T. M. Halabisky B. Wes P.. (2011). CX3CR1 protein signaling modulates microglial activation and protects against plaque-independent cognitive deficits in a mouse model of alzheimer disease. J. Biol. Chem. 286, 32713–32722. 10.1074/jbc.M111.254268 21771791 PMC3173153

[B27] Condello C. Yuan P. Schain A. Grutzendler J. (2015). Microglia constitute a barrier that prevents neurotoxic protofibrillar Aβ42 hotspots around plaques. Nat. Commun. 6:6176. 10.1038/ncomms7176 25630253 PMC4311408

[B28] Conn P. J. Jones C. K. Lindsley C. W. (2009). Subtype-selective allosteric modulators of muscarinic receptors for the treatment of CNS disorders. Trends Pharmacol. Sci. 30, 148–155. 10.1016/j.tips.2008.12.002 19201489 PMC2907736

[B29] Conn P. J. Lindsley C. W. Meiler J. Niswender C. M. (2014). Opportunities and challenges in the discovery of allosteric modulators of GPCRs for treating CNS disorders. Nat. Rev. Drug Discov. 13, 692–708. 10.1038/nrd4308 25176435 PMC4208620

[B30] Daaka Y. Luttrell L. M. Lefkowitz R. J. (1997). Switching of the coupling of the β(2)-adrenergic receptor to different G proteins by protein kinase A. Nature 390, 88–91. 10.1038/36362 9363896

[B31] Das U. Wang L. Ganguly A. Saikia J. M. Wagner S. L. Koo E. H.. (2016). Visualizing APP and BACE-1 approximation in neurons yields insight into the amyloidogenic pathway. Nat. Neurosci. 19, 55–64. 10.1038/nn.4188 26642089 PMC4782935

[B32] Davis A. A. Fritz J. J. Wess J. Lah J. J. Levey A. I. (2010). Deletion of M-1 muscarinic acetylcholine receptors increases amyloid pathology *in vitro* and *in vivo*. J. Neurosci. 30, 4190–4196. 10.1523/JNEUROSCI.6393-09.2010 20335454 PMC2855655

[B33] Doré A. S. Okrasa K. Patel J. C. Serrano-Vega M. Bennett K. Cooke R. M.. (2014). Structure of class C GPCR metabotropic glutamate receptor 5 transmembrane domain. Nature 511, 557–562. 10.1038/nature13396 25042998

[B34] El Khoury J. Toft M. Hickman S. E. Means T. K. Terada K. Geula C.. (2007). Ccr2 deficiency impairs microglial accumulation and accelerates progression of Alzheimer-like disease. Nat. Med. 13, 432–438. 10.1038/nm1555 17351623

[B35] Erb L. Cao C. Ajit D. Weisman G. A. (2015). P2Y receptors in Alzheimer’s disease. Biol. Cell 107, 1–21. 10.1111/boc.201400043 25179475 PMC4286471

[B36] Espinosa J. Rocha A. Nunes F. Costa M. S. Schein V. Kazlauckas V.. (2013). Caffeine consumption prevents memory impairment, neuronal damage and adenosine A(2A) receptors upregulation in the hippocampus of a rat model of sporadic dementia. J. Alzheimers Dis. 34, 509–518. 10.3233/JAD-111982 23241554

[B37] Fenalti G. Zatsepin N. A. Betti C. Giguere P. Han G. W. Ishchenko A.. (2015). Structural basis for bifunctional peptide recognition at human delta-opioid receptor. Nat. Struct. Mol. Biol. 22, 265–268. 10.1038/nsmb.2965 25686086 PMC4351130

[B38] Ferguson S. S. G. (2007). Phosphorylation-independent attenuation of GPCR signalling. Trends Pharmacol. Sci. 28, 173–179. 10.1016/j.tips.2007.02.008 17350109

[B39] Fisher A. Heldman E. Gurwitz D. Haring R. Barak D. Meshulam H.. (1993). Selective signaling via unique M1 muscarinic agonists. Ann. N Y Acad. Sci. 695, 300–303. 10.1111/j.1749-6632.1993.tb23070.x 8239299

[B40] Fredriksson R. Lagerström M. C. Lundin L. G. Schiöth H. B. (2003). The G-protein-coupled receptors in the human genome form five main families. Phylogenetic analysis, paralogon groups and fingerprints. Mol. Pharmacol. 63, 1256–1272. 10.1124/mol.63.6.1256 12761335

[B41] Gallagher M. King R. A. Young N. B. (1983). Opiate antagonists improve spatial memory. Science 221, 975–976. 10.1126/science.6879198 6879198

[B42] Giannoni P. Gaven F. de Bundel D. Baranger K. Marchetti-Gauthier E. Roman F. S.. (2013). Early administration of RS67333, a specific 5-HT4 receptor agonist, prevents amyloidogenesis and behavioral deficits in the 5XFAD mouse model of Alzheimer’s disease. Front. Aging Neurosci. 5:96. 10.3389/fnagi.2013.00096 24399967 PMC3871961

[B43] Ginsberg S. D. Alldred M. J. Counts S. E. Cataldo A. M. Neve R. L. Jiang Y.. (2010). Microarray analysis of hippocampal CA1 neurons implicates early endosomal dysfunction during Alzheimer’s disease progression. Biol. Psychiatry 68, 885–893. 10.1016/j.biopsych.2010.05.030 20655510 PMC2965820

[B44] Giunta S. Andriolo V. Castorina A. (2014). Dual blockade of the A(1) and A(2A) adenosine receptor prevents amyloid β toxicity in neuroblastoma cells exposed to aluminum chloride. Int. J. Biochem. Cell Biol. 54, 122–136. 10.1016/j.biocel.2014.07.009 25058312

[B45] Granier S. Manglik A. Kruse A. C. Kobilka T. S. Thian F. S. Weis W. I.. (2012). Structure of the delta-opioid receptor bound to naltrindole. Nature 485, 400–404. 10.1038/nature11111 22596164 PMC3523198

[B46] Haga K. Kruse A. C. Asada H. Yurugi-Kobayashi T. Shiroishi M. Zhang C.. (2012). Structure of the human M2 muscarinic acetylcholine receptor bound to an antagonist. Nature 482, 547–551. 10.1038/nature10753 22278061 PMC3345277

[B47] Hanson M. A. Roth C. B. Jo E. Griffith M. T. Scott F. L. Reinhart G.. (2012). Crystal structure of a lipid g protein-coupled receptor. Science 335, 851–855. 10.1126/science.1215904 22344443 PMC3338336

[B50] He X. Li F. Chang W. P. Tang J. (2005). GGA proteins mediate the recycling pathway of memapsin 2 (BACE). J. Biol. Chem. 280, 11696–11703. 10.1074/jbc.m411296200 15615712

[B49] He W. Lu Y. Qahwash I. Hu X. Y. Chang A. Yan R. (2004). Reticulon family members modulate BACE1 activity and amyloid-β peptide generation. Nat. Med. 10, 959–965. 10.1038/nm1088 15286784

[B51] Heese K. (2013). G Proteins, p60TRP and neurodegenerative diseases. Mol. Neurobiol. 47, 1103–1111. 10.1007/s12035-013-8410-1 23345134

[B52] Heese K. Nagai Y. Sawada T. (2004). Nerve growth factor (NGF) induces mRNA expression of the new transcription factor protein p48ZnF. Exp. Mol. Med. 36, 130–134. 10.1038/emm.2004.19 15150441

[B53] Henderson V. W. Roberts E. Wimer C. Bardolph E. L. Chui H. C. Damasio A. R.. (1989). Multicenter trial of naloxone in Alzheimer’s disease. Ann. Neurol. 25, 404–406. 10.1002/ana.410250413 2653175

[B54] Hinckley M. Vaccari S. Horner K. Chen R. Conti M. (2005). The G-protein-coupled receptors GPR3 and GPR12 are involved in cAMP signaling and maintenance of meiotic arrest in rodent oocytes. Dev. Biol. 287, 249–261. 10.1016/j.ydbio.2005.08.019 16229830

[B55] Hollenstein K. Kean J. Bortolato A. Cheng R. K. Y. Doré A. S. Jazayeri A.. (2013). Structure of class B GPCR corticotropin-releasing factor receptor 1. Nature 499, 438–443. 10.1038/nature12357 23863939

[B56] Hopkins A. L. Groom C. R. (2002). The druggable genome. Nat. Rev. Drug Discov. 1, 727–730. 10.1038/nrd892 12209152

[B57] Huang Y. Skwarek-Maruszewska A. Horre K. Vandewyer E. Wolfs L. Snellinx A.. (2015). Loss of GPR3 reduces the amyloid plaque burden and improves memory in Alzheimer’s disease mouse models. Sci. Transl. Med. 7:309ra164. 10.1126/scitranslmed.aab3492 26468326

[B58] Jaakola V. P. Griffith M. T. Hanson M. A. Cherezov V. Chien E. Y. T. Lane J. R.. (2008). The 2.6 angstrom crystal structure of a human A(2A) adenosine receptor bound to an antagonist. Science 322, 1211–1217. 10.1126/science.1164772 18832607 PMC2586971

[B59] Jiang S. T. Wang Y. Ma Q. L. Zhou A. N. Zhang X. Zhang Y. W. (2012). M1 muscarinic acetylcholine receptor interacts with BACE1 and regulates its proteosomal degradation. Neurosci. Lett. 515, 125–130. 10.1016/j.neulet.2012.03.026 22450048

[B60] Jones C. K. Brady A. E. Davis A. A. Xiang Z. Bubser M. Tantawy M. N.. (2008). Novel selective allosteric activator of the M1 muscarinic acetylcholine receptor regulates amyloid processing and produces antipsychotic-like activity in rats. J. Neurosci. 28, 10422–10433. 10.1523/JNEUROSCI.1850-08.2008 18842902 PMC2577155

[B61] Justice N. J. Huang L. Tian J.-B. Cole A. Pruski M. Hunt A. J. Jr.. (2015). Posttraumatic stress disorder-like induction elevates β-amyloid levels, which directly activates corticotropin-releasing factor neurons to exacerbate stress responses. J. Neurosci. 35, 2612–2623. 10.1523/JNEUROSCI.3333-14.2015 25673853 PMC4323535

[B62] Kang E. L. Biscaro B. Piazza F. Tesco G. (2012). BACE1 protein endocytosis and trafficking are differentially regulated by ubiquitination at lysine 501 and the Di-leucine motif in the carboxyl terminus. J. Biol. Chem. 287, 42867–42880. 10.1074/jbc.M112.407072 23109336 PMC3522283

[B63] Katritch V. Cherezov V. Stevens R. C. (2013). Structure-function of the G protein-coupled receptor superfamily. Annu. Rev. Pharmacol. Toxicol. 53, 531–556. 10.1146/annurev-pharmtox-032112-135923 23140243 PMC3540149

[B64] Khoury E. Clément S. Laporte S. A. (2014). Allosteric and biased g protein-coupled receptor signaling regulation: potentials for new therapeutics. Front. Endocrinol. (Lausanne) 5:68. 10.3389/fendo.2014.00068 24847311 PMC4021147

[B65] Kieffer B. L. Gavériaux-Ruff C. (2002). Exploring the opioid system by gene knockout. Prog. Neurobiol. 66, 285–306. 10.1016/s0301-0082(02)00008-4 12015197

[B66] Kim S. H. Fraser P. E. Westaway D. St George-Hyslop P. H. Ehrlich M. E. Gandy S. (2010). Group II metabotropic glutamate receptor stimulation triggers production and release of Alzheimer’s amyloid(β)42 from isolated intact nerve terminals. J. Neurosci. 30, 3870–3875. 10.1523/JNEUROSCI.4717-09.2010 20237257 PMC2857209

[B67] Kirazov L. Löffler T. Schliebs R. Bigl V. (1997). Glutamate-stimulated secretion of amyloid precursor protein from cortical rat brain slices. Neurochem. Int. 30, 557–563. 10.1016/s0197-0186(96)00119-2 9152997

[B68] Koh Y. H. von Arnim C. A. Hyman B. T. Tanzi R. E. Tesco G. (2005). BACE is degraded via the lysosomal pathway. J. Biol. Chem. 280, 32499–32504. 10.1074/jbc.m506199200 16033761

[B69] Kojro E. Postina R. Buro C. Meiringer C. Gehrig-Burger K. Fahrenholz F. (2006). The neuropeptide PACAP promotes α-secretase pathway for processing Alzheimer amyloid precursor protein. FASEB J. 20, 512–514. 10.1096/fj.05-4812fje 16401644

[B70] Krauthausen M. Kummer M. P. Zimmermann J. Reyes-Irisarri E. Terwel D. Bulic B.. (2015). CXCR3 promotes plaque formation and behavioral deficits in an Alzheimer’s disease model. J. Clin. Invest. 125, 365–378. 10.1172/JCI66771 25500888 PMC4382235

[B71] Kruse A. C. Hu J. X. Pan A. C. Arlow D. H. Rosenbaum D. M. Rosemond E.. (2012). Structure and dynamics of the M3 muscarinic acetylcholine receptor. Nature 482, 552–556. 10.1038/nature10867 22358844 PMC3529910

[B72] Langmead C. J. Watson J. Reavill C. (2008). Muscarinic acetylcholine receptors as CNS drug targets. Pharmacol. Ther. 117, 232–243. 10.1016/j.pharmthera.2007.09.009 18082893

[B75] Lee S. Varvel N. H. Konerth M. E. Xu G. Cardona A. E. Ransohoff R. M.. (2010). CX3CR1 deficiency alters microglial activation and reduces β-amyloid deposition in two Alzheimer’s disease mouse models. Am. J. Pathol. 177, 2549–2562. 10.2353/ajpath.2010.100265 20864679 PMC2966811

[B74] Lee R. K. Wurtman R. J. Cox A. J. Nitsch R. M. (1995). Amyloid precursor protein processing is stimulated by metabotropic glutamate receptors. Proc. Natl. Acad. Sci. U S A 92, 8083–8087. 10.1073/pnas.92.17.8083 7644542 PMC41291

[B73] Lee H. G. Zhu X. W. Casadesus G. Pallàs M. Carmins A. O’Neill M. J.. (2009). The effect of mGluR2 activation on signal transduction pathways and neuronal cell survival. Brain Res. 1249, 244–250. 10.1016/j.brainres.2008.10.055 19026996 PMC2698437

[B76] Lefkowitz R. J. Shenoy S. K. (2005). Transduction of receptor signals by β-arrestins. Science 308, 512–517. 10.1126/science.1109237 15845844

[B78] Liu Z. Condello C. Schain A. Harb R. Grutzendler J. (2010). CX3CR1 in microglia regulates brain amyloid deposition through selective protofibrillar amyloid-β phagocytosis. J. Neurosci. 30, 17091–17101. 10.1523/JNEUROSCI.4403-10.2010 21159979 PMC3077120

[B77] Liu X. Zhao X. Zeng X. Bossers K. Swaab D. F. Zhao J.. (2013). β-arrestin1 regulates γ-secretase complex assembly and modulates amyloid-β pathology. Cell Res. 23, 351–365. 10.1038/cr.2012.167 23208420 PMC3587707

[B79] Luttrell L. M. Maudsley S. Bohn L. M. (2015). Fulfilling the promise of ’biased’ GPCR agonism. Mol. Pharmacol. 88, 579–588. 10.1124/mol.115.099630 26134495 PMC4551052

[B80] Ly P. T. T. Wu Y. L. Zou H. Y. Wang R. T. Zhou W. H. Kinoshita A.. (2013). Inhibition of GSK3 β-mediated BACE1 expression reduces Alzheimer-associated phenotypes. J. Clin. Invest. 123, 224–235. 10.1172/JCI64516 23202730 PMC3533290

[B81] Maher-Edwards G. Zvartau-Hind M. Hunter A. J. Gold M. Hopton G. Jacobs G.. (2010). Double-blind, controlled phase II study of a 5-HT6 receptor antagonist, SB-742457, in Alzheimer’s disease. Curr. Alzheimer Res. 7, 374–385. 10.2174/156720510791383831 20043816

[B82] Manglik A. Kruse A. C. Kobilka T. S. Thian F. S. Mathiesen J. M. Sunahara R. K.. (2012). Crystal structure of the mu-opioid receptor bound to a morphinan antagonist. Nature 485, 321–326. 10.1038/nature10954 22437502 PMC3523197

[B83] Melancon B. J. Tarr J. C. Panarese J. D. Wood M. R. Lindsley C. W. (2013). Allosteric modulation of the M1 muscarinic acetylcholine receptor: improving cognition and a potential treatment for schizophrenia and Alzheimer’s disease. Drug Discov. Today 18, 1185–1199. 10.1016/j.drudis.2013.09.005 24051397 PMC3876030

[B84] Mishra M. Heese K. (2011). P60TRP interferes with the GPCR/secretase pathway to mediate neuronal survival and synaptogenesis. J. Cell. Mol. Med. 15, 2462–2477. 10.1111/j.1582-4934.2010.01248.x 21199326 PMC3822957

[B85] Murakami M. Kouyama T. (2008). Crystal structure of squid rhodopsin. Nature 453, 363–367. 10.1038/nature06925 18480818

[B86] Murayama K. S. Kametani F. Saito S. Kume H. Akiyama H. Araki W. (2006). Reticulons RTN3 and RTN4-B/C interact with BACE1 and inhibit its ability to produce amyloid β-protein. Eur. J. Neurosci. 24, 1237–1244. 10.1111/j.1460-9568.2006.05005.x 16965550

[B87] Nagano M. Toshima J. Y. Toshima J. (2015). Rab GTPases networks in membrane traffic in *Saccharomyces cerevisiae*. Yakugaku Zasshi 135, 483–492. 10.1248/yakushi.14-00246 25759056

[B88] Nagpure B. V. Bian J.-S. (2014). Hydrogen sulfide inhibits A2A adenosine receptor agonist induced β-amyloid production in SH-SY5Y neuroblastoma cells via a cAMP dependent pathway. PLoS One 9:e88508. 10.1371/journal.pone.0088508 24523906 PMC3921165

[B89] Nelson C. D. Sheng M. (2013). Gpr3 stimulates Aβ production via interactions with APP and β-arrestin2. PLoS One 8:e74680. 10.1371/journal.pone.0074680 24069330 PMC3771882

[B90] New D. C. Wong Y. H. (2007). Molecular mechanisms mediating the G protein-coupled receptor regulation of cell cycle progression. J. Mol. Signal. 2:2. 10.1186/1750-2187-2-2 17319972 PMC1808056

[B91] Ni Y. Zhao X. Bao G. Zou L. Teng L. Wang Z.. (2006). Activation of β2-adrenergic receptor stimulates γ-secretase activity and accelerates amyloid plaque formation. Nat. Med. 12, 1390–1396. 10.1038/nm1485 17115048

[B92] Nickols H. H. Conn P. J. (2014). Development of allosteric modulators of GPCRs for treatment of CNS disorders. Neurobiol. Dis. 61, 55–71. 10.1016/j.nbd.2013.09.013 24076101 PMC3875303

[B95] Nitsch R. M. Deng M. Growdon J. H. Wurtman R. J. (1996). Serotonin 5-HT2a and 5-HT2c receptors stimulate amyloid precursor protein ectodomain secretion. J. Biol. Chem. 271, 4188–4194. 10.1074/jbc.271.8.4188 8626761

[B93] Nitsch R. M. Deng A. Wurtman R. J. Growdon J. H. (1997). Metabotropic glutamate receptor subtype mGluR1 α stimulates the secretion of the amyloid β-protein precursor ectodomain. J. Neurochem. 69, 704–712. 10.1046/j.1471-4159.1997.69020704.x 9231730

[B94] Nitsch R. M. Slack B. E. Wurtman R. J. Growdon J. H. (1992). Release of Alzheimer amyloid precursor derivatives stimulated by activation of muscarinic acetylcholine receptors. Science 258, 304–307. 10.1126/science.1411529 1411529

[B96] O’Connor T. Sadleir K. R. Maus E. Velliquette R. A. Zhao J. Cole S. L.. (2008). Phosphorylation of the translation initiation factor eIF2α increases BACE1 levels and promotes amyloidogenesis. Neuron 60, 988–1009. 10.1016/j.neuron.2008.10.047 19109907 PMC2667382

[B97] Odagaki Y. Kinoshita M. Toyoshima R. (2014). Functional activation of G-proteins coupled with muscarinic acetylcholine receptors in rat brain membranes. J. Pharmacol. Sci. 125, 157–168. 10.1254/jphs.14020fp 24849282

[B98] Okada H. Zhang W. Z. Peterhoff C. Hwang J. C. Nixon R. A. Ryu S. H.. (2010). Proteomic identification of sorting nexin 6 as a negative regulator of BACE1-mediated APP processing. FASEB J. 24, 2783–2794. 10.1096/fj.09-146357 20354142 PMC2909280

[B99] Ongali B. Nicolakakis N. Tong X.-K. Aboulkassim T. Papadopoulos P. Rosa-Neto P.. (2014). Angiotensin II type 1 receptor blocker losartan prevents and rescues cerebrovascular, neuropathological and cognitive deficits in an Alzheimer’s disease model. Neurobiol. Dis. 68, 126–136. 10.1016/j.nbd.2014.04.018 24807206

[B100] Orr A. G. Hsiao E. C. Wang M. M. Ho K. Kim D. H. Wang X.. (2015). Astrocytic adenosine receptor A(2A) and G(s)-coupled signaling regulate memory. Nat. Neurosci. 18, 423–434. 10.1038/nn.3930 25622143 PMC4340760

[B101] Palczewski K. Kumasaka T. Hori T. Behnke C. A. Motoshima H. Fox B. A.. (2000). Crystal structure of rhodopsin: a G protein-coupled receptor. Science 289, 739–745. 10.1126/science.289.5480.739 10926528

[B103] Park S. H. Das B. B. Casagrande F. Tian Y. Nothnagel H. J. Chu M. N.. (2012). Structure of the chemokine receptor CXCR1 in phospholipid bilayers. Nature 491, 779–783. 10.1038/nature11580 23086146 PMC3700570

[B102] Park H. J. Ran Y. Jung J. I. Holmes O. Price A. R. Smithson L.. (2015). The stress response neuropeptide CRF increases amyloid-β production by regulating γ-secretase activity. EMBO J. 34, 1674–1686. 10.15252/embj.201488795 25964433 PMC4475401

[B104] Pfeffer S. R. (2013). Rab GTPase regulation of membrane identity. Curr. Opin. Cell Biol. 25, 414–419. 10.1016/j.ceb.2013.04.002 23639309 PMC3729790

[B105] Pimenova A. A. Thathiah A. De Strooper B. Tesseur I. (2014). Regulation of amyloid precursor protein processing by serotonin signaling. PLoS One 9:e87014. 10.1371/journal.pone.0087014 24466315 PMC3897773

[B106] Qing H. Zhou W. Christensen M. A. Sun X. Tong Y. Song W. (2004). Degradation of BACE by the ubiquitin-proteasome pathway. FASEB J. 18, 1571–1573. 10.1096/fj.04-1994fje 15289451

[B107] Rajagopal S. Rajagopal K. Lefkowitz R. J. (2010). Teaching old receptors new tricks: biasing seven-transmembrane receptors. Nat. Rev. Drug Discov. 9, 373–386. 10.1038/nrd3024 20431569 PMC2902265

[B108] Rasmussen S. G. F. Choi H.-J. Rosenbaum D. M. Kobilka T. S. Thian F. S. Edwards P. C.. (2007). Crystal structure of the human β(2) adrenergic G-protein-coupled receptor. Nature 450, 383–387. 10.1038/nature06325 17952055

[B109] Rat D. Schmitt U. Tippmann F. Dewachter I. Theunis C. Wieczerzak E.. (2011). Neuropeptide pituitary adenylate cyclase-activating polypeptide (PACAP) slows down Alzheimer’s disease-like pathology in amyloid precursor protein-transgenic mice. FASEB J. 25, 3208–3218. 10.1096/fj.10-180133 21593432 PMC3157688

[B110] Reisberg B. Ferris S. H. Anand R. Mir P. Geibel V. De Leon M. J.. (1983). Effects of naloxone in senile dementia: a double-blind trial. N. Engl. J. Med. 308, 721–722. 10.1056/nejm198303243081213 6338389

[B111] Ribeiro J. A. Sebastião A. M. (2010). Caffeine and adenosine. J. Alzheimers Dis. 20, S3–S15. 10.3233/JAD-2010-1379 20164566

[B112] Rissman R. A. Staup M. A. Lee A. R. Justice N. J. Rice K. C. Vale W.. (2012). Corticotropin-releasing factor receptor-dependent effects of repeated stress on tau phosphorylation, solubility and aggregation. Proc. Natl. Acad. Sci. U S A 109, 6277–6282. 10.1073/pnas.1203140109 22451915 PMC3341026

[B113] Ritter S. L. Hall R. A. (2009). Fine-tuning of GPCR activity by receptor-interacting proteins. Nat. Rev. Mol. Cell Biol. 10, 819–830. 10.1038/nrm2803 19935667 PMC2825052

[B114] Robert S. J. Zugaza J. L. Fischmeister R. Gardier A. M. Lezoualc’h F. (2001). The human serotonin 5-HT4 receptor regulates secretion of non-amyloidogenic precursor protein. J. Biol. Chem. 276, 44881–44888. 10.1074/jbc.m109008200 11584021

[B115] Rosenbaum D. M. Rasmussen S. G. Kobilka B. K. (2009). The structure and function of G-protein-coupled receptors. Nature 459, 356–363. 10.1038/nature08144 19458711 PMC3967846

[B116] Rossé G. Schaffhauser H. (2010). 5-HT6 receptor antagonists as potential therapeutics for cognitive impairment. Curr. Top. Med. Chem. 10, 207–221. 10.2174/156802610790411036 20166958

[B117] Salter M. W. Kalia L. V. (2004). SRC kinases: a hub for NMDA receptor regulation. Nat. Rev. Neurosci. 5, 317–328. 10.1038/nrn1368 15034556

[B118] Sannerud R. Declerck I. Peric A. Raemaekers T. Menendez G. Zhou L.. (2011). ADP ribosylation factor 6 (ARF6) controls amyloid precursor protein (APP) processing by mediating the endosomal sorting of BACE1. Proc. Natl. Acad. Sci. U S A 108, E559–E568. 10.1073/pnas.1100745108 21825135 PMC3161548

[B119] Sayas C. L. Avila J. Wandosell F. (2002a). Glycogen synthase kinase-3 is activated in neuronal cells by Gα12 and Gα13 by Rho-independent and Rho-dependent mechanisms. J. Neurosci. 22, 6863–6875. 12177184 10.1523/JNEUROSCI.22-16-06863.2002 PMC6757878

[B120] Sayas C. L. Avila J. Wandosell F. (2002b). Regulation of neuronal cytoskeleton by lysophosphatidic acid: role of GSK-3. Biochim. Biophys. Acta 1582, 144–153. 10.1016/s1388-1981(02)00149-x 12069822

[B121] Scullion G. A. Hewitt K. N. Pardon M.-C. (2013). Corticotropin-releasing factor receptor 1 activation during exposure to novelty stress protects against Alzheimer’s disease-like cognitive decline in AβPP/PS1 Mice. J. Alzheimers Dis. 34, 781–793. 10.3233/JAD-122164 23302658

[B122] Selkoe D. Kopan R. (2003). Notch and presenilin: regulated intramembrane proteolysis links development and degeneration. Annu. Rev. Neurosci. 26, 565–597. 10.1146/annurev.neuro.26.041002.131334 12730322

[B123] Shahani N. Pryor W. Swarnkar S. Kholodilov N. Thinakaran G. Burke R. E.. (2014). Rheb GTPase regulates β-secretase levels and amyloid β generation. J. Biol. Chem. 289, 5799–5808. 10.1074/jbc.M113.532713 24368770 PMC3937651

[B124] Shimamura T. Shiroishi M. Weyand S. Tsujimoto H. Winter G. Katritch V.. (2011). Structure of the human histamine H1 receptor complex with doxepin. Nature 475, 65–70. 10.1038/nature10236 21697825 PMC3131495

[B125] Sinha S. Anderson J. P. Barbour R. Basi G. S. Caccavello R. Davis D.. (1999). Purification and cloning of amyloid precursor protein β-secretase from human brain. Nature 402, 537–540. 10.1038/990114 10591214

[B126] Siu F. Y. He M. de Graaf C. Han G. W. Yang D. H. Zhang Z. Y.. (2013). Structure of the human glucagon class B G-protein-coupled receptor. Nature 499, 444–449. 10.1038/nature12393 23863937 PMC3820480

[B127] Srivastava A. Yano J. Hirozane Y. Kefala G. Gruswitz F. Snell G.. (2014). High-resolution structure of the human GPR40 receptor bound to allosteric agonist TAK-875. Nature 513, 124–127. 10.1038/nature13494 25043059

[B128] Takai Y. Sasaki T. Matozaki T. (2001). Small GTP-binding proteins. Physiol. Rev. 81, 153–208. 11152757 10.1152/physrev.2001.81.1.153

[B129] Tan Q. X. Zhu Y. Li J. Chen Z. X. Han G. W. Kufareva I.. (2013). Structure of the CCR5 chemokine receptor-HIV entry inhibitor maraviroc complex. Science 341, 1387–1390. 10.1126/science.1241475 24030490 PMC3819204

[B130] Tanaka S. Ishii K. Kasai K. Yoon S. O. Saeki Y. (2007). Neural expression of G protein-coupled receptors GPR3, GPR6 and GPR12 up-regulates cyclic AMP levels and promotes neurite outgrowth. J. Biol. Chem. 282, 10506–10515. 10.1074/jbc.m700911200 17284443

[B131] Tariot P. N. Sunderland T. Weingartner H. Murphy D. L. Cohen M. R. Cohen R. M. (1986). Naloxone and Alzheimer’s cognitive, disease. and behavioral effects of a range of doses. Arch. Gen. Psychiatry 43, 727–732. 10.1001/archpsyc.1986.01800080013002 3729666

[B132] Teng L. Zhao J. Wang F. Ma L. Pei G. (2010). A GPCR/secretase complex regulates β- and γ-secretase specificity for Aβ production and contributes to AD pathogenesis. Cell Res. 20, 138–153. 10.1038/cr.2010.3 20066010

[B133] Tesco G. Koh Y. H. Kang E. L. Cameron A. N. Das S. Sena-Esteves M.. (2007). Depletion of GGA3 stabilizes BACE and enhances β-secretase activity. Neuron 54, 721–737. 10.1016/j.neuron.2007.05.012 17553422 PMC1973166

[B134] Tesseur I. Pimenova A. A. Lo A. C. Ciesielska M. Lichtenthaler S. F. De Maeyer J. H.. (2013). Chronic 5-HT4 receptor activation decreases Aβ production and deposition in hAPP/PS1 mice. Neurobiol. Aging 34, 1779–1789. 10.1016/j.neurobiolaging.2013.01.020 23474291

[B135] Thathiah A. De Strooper B. (2009). G protein-coupled receptors, cholinergic dysfunction and Aβ toxicity in Alzheimer’s disease. Sci. Signal. 2:re8. 10.1126/scisignal.293re8 19843960

[B136] Thathiah A. De Strooper B. (2011). The role of G protein-coupled receptors in the pathology of Alzheimer’s disease. Nat. Rev. Neurosci. 12, 73–87. 10.1038/nrn2977 21248787

[B137] Thathiah A. Horré K. Snellinx A. Vandewyer E. Huang Y. Ciesielska M.. (2013). β-arrestin 2 regulates Aβ generation and γ-secretase activity in Alzheimer’s disease. Nat. Med. 19, 43–49. 10.1038/nm.3023 23202293

[B138] Thathiah A. Spittaels K. Hoffmann M. Staes M. Cohen A. Horré K.. (2009). The orphan G protein-coupled receptor 3 modulates amyloid-β peptide generation in neurons. Science 323, 946–951. 10.1126/science.1160649 19213921

[B139] Thompson A. A. Liu W. Chun E. Katritch V. Wu H. X. Vardy E.. (2012). Structure of the nociceptin/orphanin FQ receptor in complex with a peptide mimetic. Nature 485, 395–399. 10.1038/nature11085 22596163 PMC3356928

[B140] Udayar V. Buggia-Prévot V. Guerreiro R. L. Siegel G. Rambabu N. Soohoo A. L.. (2013). A paired RNAi and RabGAP overexpression screen identifies Rab11 as a regulator of β-amyloid production. Cell Rep. 5, 1536–1551. 10.1016/j.celrep.2013.12.005 24373285 PMC4004174

[B141] Uhlenbrock K. Gassenhuber H. Kostenis E. (2002). Sphingosine 1-phosphate is a ligand of the human gpr3, gpr6 and gpr12 family of constitutively active G protein-coupled receptors. Cell Signal. 14, 941–953. 10.1016/s0898-6568(02)00041-4 12220620

[B142] Upton N. Chuang T. T. Hunter A. J. Virley D. J. (2008). 5-HT6 receptor antagonists as novel cognitive enhancing agents for Alzheimer’s disease. Neurotherapeutics 5, 458–469. 10.1016/j.nurt.2008.05.008 18625457 PMC5084247

[B143] Vassar R. (2014). BACE1 inhibitor drugs in clinical trials for Alzheimer’s disease. Alzheimers Res. Ther. 6:89. 10.1186/s13195-014-0089-7 25621019 PMC4304279

[B144] Venkatakrishnan A. J. Deupi X. Lebon G. Tate C. G. Schertler G. F. Babu M. M. (2013). Molecular signatures of G-protein-coupled receptors. Nature 494, 185–194. 10.1038/nature11896 23407534

[B145] Violin J. D. Crombie A. L. Soergel D. G. Lark M. W. (2014). Biased ligands at G-protein-coupled receptors: promise and progress. Trends Pharmacol. Sci. 35, 308–316. 10.1016/j.tips.2014.04.007 24878326

[B146] Wacker D. Wang C. Katritch V. Han G. W. Huang X. P. Vardy E.. (2013). Structural features for functional selectivity at serotonin receptors. Science 340, 615–619. 10.1126/science.1232808 23519215 PMC3644390

[B148] Wang C. Jiang Y. Ma J. M. Wu H. X. Wacker D. Katritch V.. (2013a). Structural basis for molecular recognition at serotonin receptors. Science 340, 610–614. 10.1126/science.1232807 23519210 PMC3644373

[B147] Wang C. Wu H. X. Katritch V. Han G. W. Huang X. P. Liu W.. (2013b). Structure of the human smoothened receptor bound to an antitumour agent. Nature 497, 338–343. 10.1038/nature12167 23636324 PMC3657389

[B149] Wang R. Ying Z. X. Zhao J. Zhang Y. Y. Wang R. Lu H.. (2012). Lys(203) and lys(382) are essential for the proteasomal degradation of BACE1. Curr. Alzheimer Res. 9, 606–615. 10.2174/156720512800618026 22299711

[B150] Warne T. Serrano-Vega M. J. Baker J. G. Moukhametzianov R. Edwards P. C. Henderson R.. (2008). Structure of a β_1_-adrenergic G-protein-coupled receptor. Nature 454, 486–491. 10.1038/nature07101 18594507 PMC2923055

[B151] Wei H. Ahn S. Shenoy S. K. Karnik S. S. Hunyady L. Luttrell L. M.. (2003). Independent β-arrestin 2 and G protein-mediated pathways for angiotensin II activation of extracellular signal-regulated kinases 1 and 2. Proc. Natl. Acad. Sci. U S A 100, 10782–10787. 10.1073/pnas.1834556100 12949261 PMC196880

[B152] Wess J. Eglen R. M. Gautam D. (2007). Muscarinic acetylcholine receptors: mutant mice provide new insights for drug development. Nat. Rev. Drug Discov. 6, 721–733. 10.1038/nrd2379 17762886

[B153] White J. F. Noinaj N. Shibata Y. Love J. Kloss B. Xu F.. (2012). Structure of the agonist-bound neurotensin receptor. Nature 490, 508–513. 10.1038/nature11558 23051748 PMC3482300

[B154] Wisely E. V. Xiang Y. K. Oddo S. (2014). Genetic suppression of β2-adrenergic receptors ameliorates tau pathology in a mouse model of tauopathies. Hum. Mol. Genet. 23, 4024–4034. 10.1093/hmg/ddu116 24626633 PMC4082366

[B155] Wu B. L. Chien E. Y. T. Mol C. D. Fenalti G. Liu W. Katritch V.. (2010). Structures of the CXCR4 chemokine GPCR with small-molecule and cyclic peptide antagonists. Science 330, 1066–1071. 10.1126/science.1194396 20929726 PMC3074590

[B156] Wu H. X. Wacker D. Mileni M. Katritch V. Han G. W. Vardy E.. (2012). Structure of the human κ-opioid receptor in complex with JDTic. Nature 485, 327–332. 10.1038/nature10939 22437504 PMC3356457

[B157] Wu H. Wang C. Gregory K. J. Han G. W. Cho H. P. Xia Y.. (2014). Structure of a class C GPCR metabotropic glutamate receptor 1 bound to an allosteric modulator. Science 344, 58–64. 10.1126/science.1249489 24603153 PMC3991565

[B48] Yan R. Vassar R. (2014). Targeting the β secretase BACE1 for Alzheimer’s disease therapy. Lancet Neurol. 13, 319–329. 10.1016/S1474-4422(13)70276-X 24556009 10.1016/S1474-4422(13)70276-X PMC4086426

[B159] Yang R. Jiang X. Ji R. Meng L. Liu F. Chen X.. (2015). Therapeutic potential of PACAP for neurodegenerative diseases. Cell. Mol. Biol. Lett. 20, 265–278. 10.1515/cmble-2015-0008 26204407

[B158] Yang L. B. Lindholm K. Yan R. Q. Citron M. Xia W. M. Yang X. L.. (2003). Elevated β-secretase expression and enzymatic activity detected in sporadic Alzheimer disease. Nat. Med. 9, 3–4. 10.1038/nm0103-3 12514700

[B160] Yin J. Mobarec J. C. Kolb P. Rosenbaum D. M. (2015). Crystal structure of the human OX2 orexin receptor bound to the insomnia drug suvorexant. Nature 519, 247–250. 10.1038/nature14035 25533960

[B161] Zeitlin R. Patel S. Burgess S. Arendash G. W. Echeverria V. (2011). Caffeine induces beneficial changes in PKA signaling and JNK and ERK activities in the striatum and cortex of Alzheimer’s transgenic mice. Brain Res. 1417, 127–136. 10.1016/j.brainres.2011.08.036 21907331

[B166] Zhang M. Deng Y. Luo Y. Zhang S. Zou H. Cai F.. (2012a). Control of BACE1 degradation and APP processing by ubiquitin carboxyl-terminal hydrolase L1. J. Neurochem. 120, 1129–1138. 10.1111/j.1471-4159.2011.07644.x 22212137

[B163] Zhang D. Gao Z.-G. Zhang K. Kiselev E. Crane S. Wang J.. (2015a). Two disparate ligand-binding sites in the human P2Y(1) receptor. Nature 520, 317–321. 10.1038/nature14287 25822790 PMC4408927

[B162] Zhang C. Srinivasan Y. Arlow D. H. Fung J. J. Palmer D. Zheng Y. W.. (2012b). High-resolution crystal structure of human protease-activated receptor 1. Nature 492, 387–392. 10.1038/nature11701 23222541 PMC3531875

[B164] Zhang H. Unal H. Gati C. Han G. W. Liu W. Zatsepin N. A.. (2015b). Structure of the Angiotensin receptor revealed by serial femtosecond crystallography. Cell 161, 833–844. 10.1016/j.cell.2015.04.011 25913193 PMC4427029

[B165] Zhang K. Zhang J. Gao Z. G. Zhang D. Zhu L. Han G. W.. (2014). Structure of the human P2Y12 receptor in complex with an antithrombotic drug. Nature 509, 115–118. 10.1038/nature13083 24670650 PMC4174307

[B167] Zhou X. B. Wulfsen I. Lutz S. Utku E. Sausbier U. Ruth P.. (2008). M2 muscarinic receptors induce airway smooth muscle activation via a dual, Gβγ-mediated inhibition of large conductance Ca2+-activated K+ channel activity. J. Biol. Chem. 283, 21036–21044. 10.1074/jbc.m800447200 18524769 PMC3258941

[B168] Züchner T. Perez-Polo J. R. Schliebs R. (2004). β-secretase BACE1 is differentially controlled through muscarinic acetylcholine receptor signaling. J. Neurosci. Res. 77, 250–257. 10.1002/jnr.20152 15211591

